# Application of RNAi-induced gene expression profiles for prognostic prediction in breast cancer

**DOI:** 10.1186/s13073-016-0363-3

**Published:** 2016-10-27

**Authors:** Yue Wang, Kenneth M. K. Mark, Matthew H. Ung, Arminja Kettenbach, Todd Miller, Wei Xu, Wenqing Cheng, Tian Xia, Chao Cheng

**Affiliations:** 1 School of Electronic Information and Communications, Huazhong University of Science and Technology, Wuhan, Hubei 430074 China; 2 Department of Molecular and Systems Biology, Geisel School of Medicine at Dartmouth, Hanover, NH 03755 USA; 3 Norris Cotton Cancer Center, Geisel School of Medicine at Dartmouth, Lebanon, NH 03766 USA; 4 Department of Biochemistry, Geisel School of Medicine at Dartmouth, Hanover, NH 03755 USA; 5 Department of Biomedical Data Sciences, Geisel School of Medicine at Dartmouth, Lebanon, NH 03766 USA

**Keywords:** Homologous recombination pathway, Gene knockdown profiles, Cell proliferation, Cancer prognosis, Neoadjuvant chemotherapy, Genomic instability

## Abstract

Homologous recombination (HR) is the primary pathway for repairing double-strand DNA breaks implicating in the development of cancer. RNAi-based knockdowns of *BRCA1* and *RAD51* in this pathway have been performed to investigate the resulting transcriptomic profiles. Here we propose a computational framework to utilize these profiles to calculate a score, named RNA-Interference derived Proliferation Score (RIPS), which reflects cell proliferation ability in individual breast tumors. RIPS is predictive of breast cancer classes, prognosis, genome instability, and neoadjuvant chemosensitivity. This framework directly translates the readout of knockdown experiments into potential clinical applications and generates a robust biomarker in breast cancer.

**Electronic supplementary material:**

The online version of this article (doi:10.1186/s13073-016-0363-3) contains supplementary material, which is available to authorized users.

## Background

Homologous recombination (HR) is the primary pathway for repairing double-strand DNA breaks (DSBs) and is highly conserved in eukaryotic organisms [1, 2]. *BRCA1* and *BRCA2*, the most well-studied genes in this pathway [3, 4], participate in DNA repair either as essential proteins themselves or regulate other proteins in the HR pathway to facilitate repair of damaged DNA or apoptosis if DNA cannot be repaired [5]. BRCA1 and BRCA2 interact with RAD51 to form complexes that initiate and facilitate homologous recombination [6–9]. RAD51 catalyzes the key reactions in the HR pathway, including homology search and DNA strand invasion [1].

Mutation or deregulation of genes in the HR pathway has been implicated in the development of many cancer subtypes including breast cancer [10]. For example, mutations in *BRCA1* and *BRCA2* have long been known to confer cancer susceptibility [11] and women with a germ-line heterozygous mutation have an overall increased lifetime risk of developing breast and ovarian cancers [12, 13]. Although the majority of breast tumors are sporadic and do not carry germline mutations in *BRCA1* or *BRCA2* [14], it has been shown that the HR DNA repair pathway is frequently disrupted by numerous mechanisms [13, 15]. For example, methylation of the *BRCA1* promoter has been shown to transcriptionally silence *BRCA1* [16] leading to lower levels of *BRCA1* messenger RNA (mRNA), which correlates with disease characteristics of breast and ovarian cancers [17]. When functional BRCA1 or BRCA2 are absent or dysfunctional and unable to perform HR-mediated repair of double-strand DNA breaks, alternative error-prone pathways, such as non-homologous end joining and single-strand annealing are induced, leading to a significant increase in genome instability [18]. In addition, many drugs have recently been developed to exploit the role of the HR pathway in cancer development [10] and HR pathway activity has been implicated in cancer treatment and drug resistance [10, 19].

Recently, the concept of “BRCAness” has been introduced to investigate sporadic breast cancers with defects in the HR-mediated DNA repair pathway, which endow them with critical features also observed in hereditary breast cancers carrying *BRCA1* or *BRCA2* germline mutation [13, 20]. Genomic features have been selected to infer BRCAness by comparing *BRCA1/2* mutant samples with hereditary breast tumor samples [21–26]. An alternative strategy is to investigate genes that are regulated by the HR pathway. These genes can be systematically identified by using RNAi to knockdown the key genes in this pathway. Peng et al. [27] utilized MCF-10A immortalized mammary epithelial cells to build a gene signature composed of the differentially expressed genes common among three different single-gene (*BRCA1*, *RAD51*, and *BRIT1*) knockdown experiments. This signature was used in breast tumor samples to predict patients with HR defects and their overall clinical outcome. However, their signature contained just a small set of the most differentially expressed genes, rather than fully utilizing the knockdown profiles, potentially introducing bias.

In this study, we proposed a novel computational framework to investigate the similarity between the gene expression profiles derived from RNAi experiments and breast tumors gene expression profiles. In particular, we used the complete knockdown profiles generated by Peng et al. [27] to infer whether the gene expression of breast tumors from six different breast cancer datasets are similar with the knockdown profiles. These profiles contained information about every gene that is directly or indirectly regulated by the HR pathway when *BRCA1* or *RAD51* is knocked down. In contrast to the signature-based method [27], using the complete knockdown profile ensures a higher sensitivity. We described the utility of integrating knockdown gene expression profiles with a rank-based algorithm called BASE [28] and demonstrated its ability to estimate similarity between an individual patient’s baseline gene expression profile and the knockdown profile. Our results indicated that patients stratified by knockdown profiles have significant differences in terms of their breast cancer classification, prognosis, genome instability, and neoadjuvant chemosensitivity.

## Methods

### Datasets

The gene expression data for *BRCA1* and *RAD51* knockdown were generated by Peng et al. [27] and downloaded from the Gene Expression Omnibus (GEO) database [29] with accession ID GSE54266. The data contained gene expression profiles for MCF-10A that were transfected by shRNA control or shRNA designed to knockdown *BRCA1* or *RAD51*. Expression profiles replicated were averaged to obtain three profiles for control, *BRCA1* knockdown, and *RAD51* knockdown, respectively.

A total of six breast cancer datasets were used in this study as summarized in Additional file 1: Table S1. The Larsen dataset (GSE40115) contains gene expression profiles for 275 breast cancer samples, including 128 from sporadic cases, as well as 33 with BRCA1 and 22 with *BRCA2* germ-line mutations [30]. The METABRIC dataset was downloaded from the European Genome Phenome Archive with accession ID EGAS00000000083, containing profiles for 144 normal breast and 1992 tumor samples [31]. The Ur-Rehman dataset (GSE47561) is metadata that combined samples with 10 datasets, in which 1170 samples with known relapse-free survival were used in our analysis [32]. The Vijver dataset was downloaded from the Netherlands Cancer Institute (http://ccb.nki.nl/data/) [33], containing 295 breast cancer samples. The Hatzis dataset (GSE25066) contains gene expression profiles for 508 *HER2*-negative invasive breast tumor samples that were collected by fine needle aspiration (FNA) or core biopsy (CBX) prior to any systemic therapy. Patients were then treated by neoadjuvant taxane-anthracycline chemotherapy and followed to assess the treatment efficacy (pathological complete response or residual disease) [34]. The TCGA breast cancer dataset was downloaded from TCGA Data Portal website (http://tcga-data.nci.nih.gov/tcga/) [35]. The genes included in these six breast cancer datasets showed high consistency (Additional file 1: Figure S1).

### Calculation of RIPS

The knockdown profiles manifested downstream genes that were directly or indirectly regulated by the HR pathway when knocking down *BRCA1* or *RAD51*. By integrating the knockdown and baseline tumor expression profiles, we developed a novel computational method to examine the similarity between the baseline expression levels of HR pathway regulated genes and tumor. The calculation was performed as the following steps.

In the first step, we compared the expression levels of all genes in *BRCA1*/*RAD51* knockdown versus control to obtain a vector *f* = {f_1_, f_2_, …, f_n_}, where n was the total number of genes and f_i_ was the log ratio expression of gene *i* in *BRCA1*/*RAD51* knockdown versus control. Following that, we standardized the vector *f* by subtracting the mean and then divided by the standard deviation of log ratios, resulting in a vector of z-scores, *z* = {z_1_, z_2_, …, z_n_}. We then split the *z* vector into two vectors *z* ^+^ and *z* ^–^. In *z* ^+^, all positive values (upregulated genes) were preserved while negative values (downregulated genes) were replaced by 0. Similarly, in *z* ^–^, all negative values were preserved while positive values were replaced by 0. Each z_i_ corresponded to a *p* value (p_i_) referring to the standard normal distribution. Finally, we obtained two weight vectors *w* ^+^ and *w* ^–^, in which w_i_ was calculated as –log10(p_i_) followed by trimming (to avoid extreme values, we used 10 as a cutoff to trim the –log10(p_i_)) and rescaling so that w_i_ takes a value within [0, 1]. The vectors *w* ^+^ and *w* ^–^ assigned weights to upregulated and downregulated genes by the *BRCA1*/*RAD51* knockdown event. In *w* ^+^, a higher value (i.e. close to 1) indicated higher upregulation and w^+^_i_ = 0 for all downregulated genes. Conversely, in *w* ^–^ a higher value indicates higher downregulation and w^-^_i_ = 0 for all upregulated genes.

In the second step, we processed breast cancer gene expression data to obtain relative expression levels of genes in each sample. Gene expression data measured by one-channel array platforms represent absolute expression level of genes. We transformed the values into log scale and then performed quantile normalization [36] at the gene level so that all samples in a dataset have a matching distribution. Then for each gene, we converted its values into relative expression levels by subtracting its median expression over all samples. For gene expression data measured by two-channel array platforms, no processing was required, since gene expression levels were already represented as relative values (log ratios). Eventually, we obtained a gene expression matrix that contained relative expression levels of all genes in all samples.

In the third step, given the gene expression profile for a breast cancer sample (*e* = {e_1_, e_2_,…, e_n_}) and the two weight vectors (*w* ^+^ = {w^+^_1_, w^+^_2_,...., w^+^_n_} and *w* ^–^ = {w^–^_1_, w^–^_2_,...., w^–^_n_}), we applied a modified version of a statistical method called BASE [28] to calculate the similarity score in this sample. Briefly, genes in *e*, *w* ^+^, and *w* ^–^ were reordered so that the relative expression of genes decreased monotonically. Then two CDF (cumulative distribution function) -like functions, denoted as h(i) and b(i), were defined to quantify the correlation between vector *e* and vector *w* (*w* ^+^ or *w* ^–^). When highly expressed genes tend to have higher weight in vector *w*, h(i) increases rapidly and b(i) increases slowly. The opposite will be observed if highly expressed genes tend to have lower weight in vector *w*. Thus, the maximum deviation between h(i) and b(i) could be used to represent the biased distribution of HR-regulated genes (genes with high weight) in the expression profile for a sample. After normalizing the resulting maximum deviation against an empirical null distribution generated by permutations, we obtained two scores, score^+^ and score^–^, based on *w* ^+^, and *w* ^–^, respectively.

Finally, we combined the two scores to obtain a similarity score = score^+^ – score^–^. The resulting scores measure the similarity between the expression profiles of these tumor samples with the knockdown profile for *BRCA1* or *RAD51*. The higher the scores, the more similar a tumor’s baseline expression is to the expression of knockdown *BRCA1* or *RAD51*. Furthermore, we found that the calculated similarity score reflects the ability of cell proliferation (see “Results” for details). Therefore, we took the negative of the similarity score calculated by our method, and defined it as the RNA-Interference derived Proliferation Score (RIPS), where a low RIPS indicates high similarly to cell cycle arrest causing by the knockdown profile and thus low cellular proliferation.

### Survival analysis

The Cox proportional hazards model, Wald test, was used to investigate the relationships between the RIPS and patient survival while considering important clinical factors such as age and tumor stage. To compare survival times of patients in different groups, the Kaplan–Meier method, log-rank test, was used to estimate the survival functions of each group. The R package “survival” was used to implement survival analysis.

### Random forest model to predict pathologic complete response versus residual disease classes

Random forest models [37] were used to predict the classes, pathologic complete response (pCR) versus residual disease (RD), in the Hatzis dataset. In one model, only clinical factors were used as predictors, including age, ER status, PR status, HER2 status, stage, grade, and node status. In another model, RIPSs for sample were included as an additional predictor together with clinical factors. Prediction accuracy of models was measured by AUC scores and evaluated by using tenfold cross-validation. For each model, evaluation was performed ten times and the average AUC score was used to represent the accuracy. The R package “RandomForest” was used to implement these models.

### Measurement of TCGA genome instability

Three metrics were used to measure the genome stability of breast cancer samples from TCGA [35]. First, the total number of somatic mutated genes in samples was determined based on DNA-sequencing data. A Mutation Annotation File was downloaded from the Broad Institute’s GDAC Firehose Pipeline [38]. We summed and recorded the number of genes that had at least one non-synonymous or indel mutation for each patient, representing an absolute count of somatic mutations within that tumor. Second, copy number deviation (CND) was calculated based on copy number variation data of samples by using the following equation:

$$ CND=\frac{{\displaystyle {\sum}_{i=1}^k}\left|{ \log}_2\left({c}_i/2\right)*{f}_i\right|}{N}, $$

where c_i_ and f_i_ were the copy number and the size of DNA segment *i*, *k* was the total number of abnormal segments called by TCGA, and *N* is the size of human genome. This value measured the deviation of a cancer genome from normal in terms of copy number, with a value of 0 indicated a completely normal genome. A high CND indicated more deletions or duplications for regions of the genome. Copy number variation segment files for breast cancer samples were downloaded from TCGA portal [35]. Finally, we utilized data containing an estimate of the “ploidy” for each TCGA sample (which were calculated using the ABSOLUTE algorithm [39]) and downloaded this file from [40].

### Gene functional annotation analysis

Gene functional annotation analysis was performed to identify pathways that were enriched in the upregulated and downregulated genes by *BRCA1* or *RAD51* knockdown. Upregulated and downregulated genes were identified as the 300 most increased or decreased expression genes, respectively, in the *BRCA1*/*RAD51* knockdown with respect to the control expression profile in the GSE54266 dataset [29]. The web-based DAVID functional annotation tool (http://david.abcc.ncifcrf.gov/) was used to perform pathway enrichment analyses.

## Results

### Overview of analyses in this study

**Fig. 1:**
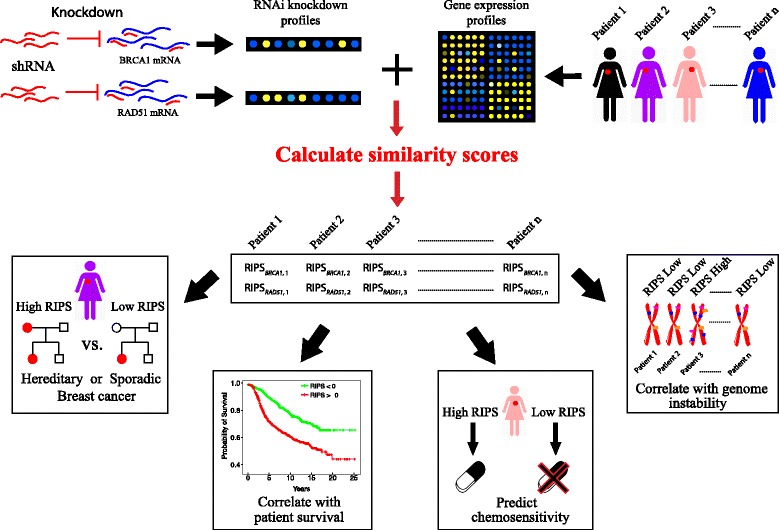
Overview of computational analysis in this study. Briefly, we utilize knockdown experiments of *BRCA1* or *RAD51* in combination with breast cancer patient gene expression data to estimate the similarity score between the knockdown profiles and tumor expression profiles. The score can be used to predict sporadic cancers from hereditary cancers, predict patient survival outcome, predict chemosensitivity, and correlate with genome instability

Our computational framework began by calculating RIPS between either *BRCA1* or *RAD51* knockdown profiles and the expression profiles of individual breast tumors as shown in Fig. 1. To carry out computation of RIPS, we used the BASE algorithm [28], which integrates gene knockdown profiles with tumor gene expression profiles. The calculated RIPS was then correlated with different genomic and pathological characteristics. First, by applying the RIPSs, we were able to discriminate with fairly high accuracy breast tumors containing germline *BRCA1* or *BRCA2* mutations from those that occurred sporadically. Second, we showed that RIPS is highly predictive of prognosis among breast cancer patients. Third, we demonstrated that treatment efficacy of neoadjuvant chemotherapy can be accurately predicted using RIPS. Finally, we estimated the genomic instability and DNA methylation levels of breast cancer samples using RIPS as predictor.

### Comparison between hereditary and sporadic breast cancer samples

We first examined the functional categories of genes regulated by *BRCA1* or *RAD51* identified from their knockdown profiles. As a result, we identified 545 upregulated and 651 downregulated genes resulting from *BRCA1* knockdown and 643 upregulated and 740 downregulated genes resulting from *RAD51* knockdown (Additional file 1: Table S2). The pathway enrichment results showed that the genes downregulated by *BRCA1* or *RAD51* are highly enriched in DNA replication, cell cycle, and mismatch repair pathways (Additional file 1: Table S3). This suggested that knockdown of *BRCA1* or *RAD51* affects cell proliferation, consistent with their functions in protecting the genome from double-strand DNA breaks during DNA replication [4, 41].

To further test the interpretation of knocking down *BRCA1* and *RAD51*, we then applied our method to calculate the similarity scores using gene expression data published by Larsen et al., which contains 33 germ-line *BRCA1* mutations, 22 germ-line *BRCA2* mutations, and 128 non-familial sporadic cancers [30]. The resulting similarity scores measured the similarity between the expression profiles of these tumor samples with the knockdown profiles. The higher the similarity score, the more similar a tumor’s baseline expression is to the expression of knockdown profile. Interestingly, we observed lower scores for hereditary samples compared with sporadic ones, although hereditary samples carry *BRCA1*/*2* mutations and are associated with defective HR pathway. Further analyses indicated these similarity scores were negatively correlated with the expression of *MKI67*, a well-known proliferation marker [42] (R = –0.81 and R = –0.78 for *BRCA1* and *RAD51*, respectively). This result indicates that the knockdown profiles of *BRCA1* and *RAD51* recapitulate the slowed proliferation characteristic of RNAi treated cells which is consistent with the pathway enrichment results (Additional file 1: Table S3). Therefore, we took the negative of the calculated similarity score and defined it as RIPS. A low RIPS indicates high similarity to the knockdown profile inferring to the low cellular proliferation.

**Fig. 2:**
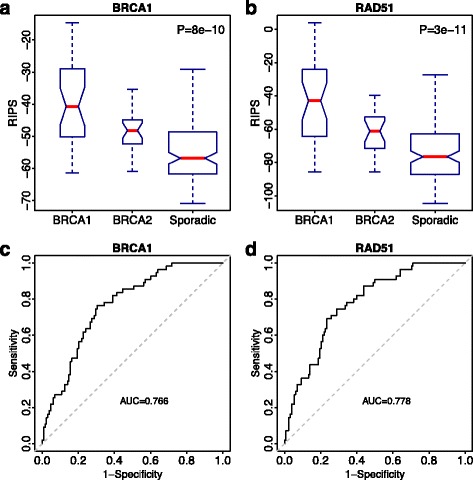
Inherited and sporadic breast cancer samples are distinguished by RIPS. **a** *Boxplot* of *BRCA1*-inherited, *BRCA2-*inherited cancers, and non-familial sporadic cancer patient RIPS scores (*BRCA1* based; RIPS _BRCA1_). The width of each *box* is proportional to the sample number. The *p* value is a calculation of one-way analysis of variance (ANOVA). **b** Same as (**a**) but utilizing RIPS _RAD51_. **c** A *ROC curve* from predicting sporadic cancers from inherited cancers using only RIPS _BRCA1_. **d** Same as (**c**) but using RIPS _RAD51_

Next, we calculated RIPS for individual tumors in the Larsen et al. [30] dataset using the knockdown profile corresponding to either *BRCA1* (Fig. 2a) or *RAD51* (Fig. 2b). In both cases, the sporadic group had significantly lower RIPSs than both the inherited *BRCA1*-mutant and *BRCA2*-mutant groups (*p* = 8e-10 versus RIPS_BRCA1_ and *p* = 8e-11 versus RIPS_RAD51_ by Mann–Whitney U-test) and the combined *BRCA1*/*BRCA2* group (Additional file 1: Figure S2). This might be due to the defective germline *BRCA1* or *BRCA2* causing more mutations resulting in higher cell proliferation. In addition, we noted that based on both RIPS_BRCA1_ and RIPS_RAD51_, patients with *BRCA1*-mutant had higher cell proliferation which might due to stronger mutation effects of *BRCA1* compared with *BRCA2* [43].

Since we observed significant differences in RIPS distribution between sporadic and inherited cancers (Fig. 2a and b), we postulated that RIPS could classify tumors as either sporadic breast cancers or cancers with germline mutations. Thus, we evaluated the sensitivity and specificity of the classification by generating a receiver operating characteristic (ROC) curve when using RIPS_BRCA1_ (Fig. 2c) and RIPS_RAD51_ (Fig. 2d) as separate features. RIPS_RAD51_ was more accurate in predicting sporadic cancers from familial cancers with an area under the curve (AUC) of 0.778, compared to an AUC of 0.766 yielded by RIPS_BRCA1_.

These observations indicate that RIPS is able to effectively distinguish sporadic breast cancers from inherited ones. In addition, the RIPS_RAD51_ not only had a higher AUC but also better distinguished between sporadic versus inherited (Fig. 2a and b), which led us to conclude that RIPS_RAD51_ is a more sensitive measure of the level of cell proliferation in a breast tumor.

### RIPS is prognostic in breast cancer

**Fig. 3:**
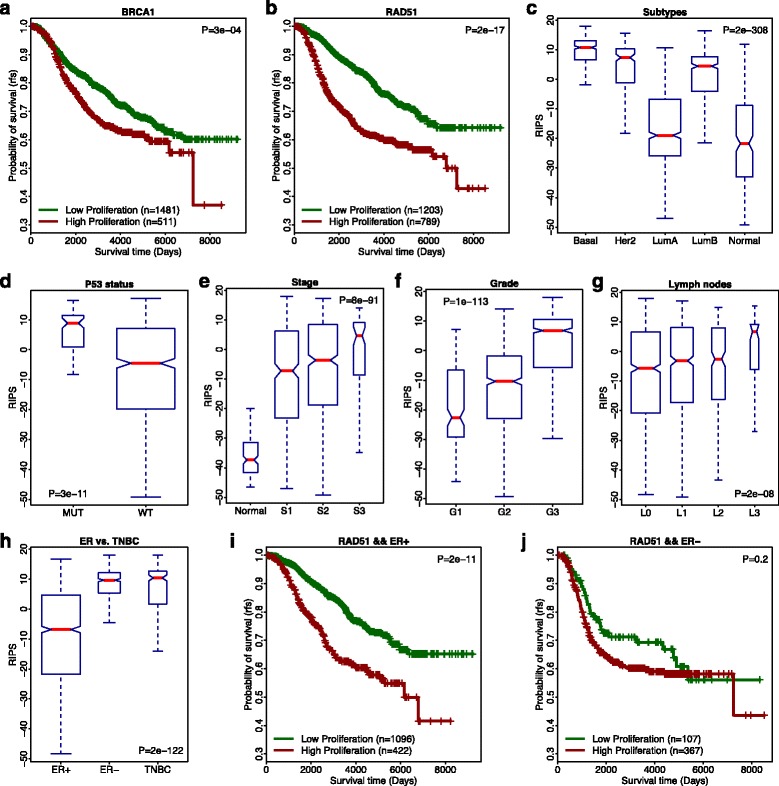
Analyses of METABRIC breast cancer patients using RIPS. **a** *Kaplan–Meier plot* using RIPS _BRCA1_. Patients with low cell proliferation (RIPS < 0; *green curve*) have a higher survival likelihood than patients with high cell proliferation (RIPS > 0; *red curve*). **b** *Kaplan–Meier plot* using RIPS_RAD51_. **c** *Boxplot* of RIPS_RAD51_ comparing samples across molecular subtypes. The width of each box is proportional to the sample number. The *p* value is a calculation of one-way analysis of variance (ANOVA). **d** *Boxplot* of RIPS_RAD51_ comparing p53 mutant to WT-p53 samples. **e** *Boxplot* of RIPS_RAD51_ comparing samples across tumor stages. **f** *Boxplot* of RIPS_RAD51_ comparing samples across lymph nodes status. **g** *Boxplot* of RIPS_RAD51_ comparing samples across tumor grades. **h** *Boxplot* of RIPS_RAD51_ comparing ER+, ER–, and TNBC samples. **i** *Kaplan–Meier plot* using RIPS_RAD51_ for only ER+ patients. **j** *Kaplan–Meier plot* using RIPS_RAD51_ for only ER– patients

As cellular proliferation is highly important in breast cancer development and progression, we tested whether RIPS is prognostic in breast cancer patients. We applied our method to the METABRIC dataset generated by Curtis et al. [31], which represents the most comprehensive breast cancer dataset to date containing gene expression profiles for 144 normal breast samples and 1992 primary breast tumor samples with detailed clinical information. For each tumor in this dataset, we calculated RIPS_BRCA1_ and RIPS_RAD51_, which were used to assign tumor samples to high (RIPS > 0) or low (RIPS < 0) RIPS groups. As shown in Fig. 3a and b, both the *BRCA1*-based and *RAD51*-based stratifications resulted in groups with significantly different relapse-free survival (RFS) times. Patients inferred to have a lower cell proliferation demonstrated a more favorable prognosis than those with higher inferred proliferation. Again, RFS survival analysis suggests that RIPS_RAD51_ is superior to RIPS_BRCA1_ for inferring cell proliferation of tumor. Based on the above results, and for simplification, we used RIPS_RAD51_ alone for subsequent analyses.

With the RIPS_RAD51_ profile, we then investigated the level of cell proliferation in breast cancer subtypes stratified by molecular subtype, *TP53* status, cancer stage, cancer grade, lymph node status, and histopathological subtype. We first examined the RIPSs in the five breast cancer molecular subtypes: Basal-like, HER2-enriched, Luminal A, Luminal B, and Normal-like. As shown in Fig. 3c, we observed a high level of cell proliferation in the Basal and HER2-enriched subtypes, a low level of proliferation in the Luminal A and Normal-like subtypes, and an intermediate level of proliferation in the Luminal B subtype (Mann–Whitney U-test, *p* = 2e-308). This indicates that Luminal A and Normal-like cancer samples have lower cell proliferation than the other three subtypes.

In the METABRIC dataset, *TP53* status was available for 820 tumors, of which 99 harbored *TP53* missense or truncating mutations. Breast tumors with *TP53* mutations had higher RIPS compared to wild-type samples (Mann–Whitney U-test, *p* = 6e-11; Fig. 3d). In addition, we observed in the METABRIC dataset that the frequency of *TP53* mutations increased as RIPS increased (Additional file 1: Figure S3A). Specifically, only 25 out of 478 samples in the RIPS-negative (RIPS < 0) group carried *TP53* mutations compared to 74 out of the 342 RIPS-positve (RIPS > 0) group, yielding a 4.1-fold difference in *TP53* mutation frequency (*p* = 3e-12, Chi-square test). Similar results were also observed in the TCGA breast cancer dataset [35] (Additional file 1: Figure S3B).

In addition, we compared RIPS of samples from normal breasts (144 samples) with breast tumors at stages 1, 2, or 3 (stage 4 samples were excluded due to limited number). RIPS was relatively low in normal breast samples and increased dramatically with increasing tumor stage (Mann–Whitney U-test, *p* = 8e-91; Fig. 3e). Similarly, RIPS increased significantly with increasing tumor grade (Mann–Whitney U-test, *p* = 1e-113; Fig. 3f) and the number of lymph nodes (Mann–Whitney U-test, *p* = 2e-08; Fig. 3g). Here, lymph node status was determined by lymph node count: L0 (no positive nodes), L1 (1–3 positive nodes), L2 (4–9 positive nodes), and L3 (>10 nodes) [44].

Moreover, our results indicated that ER+ tumors had lower RIPS than ER– and triple-negative (TNBC) tumors (Mann–Whitney U-test, *p* = 2e-122; Fig. 3h). As a consequence, we examined the prognostic predictive power of RIPS in patients with ER+ and ER– tumors separately. We found that RIPS predicted RFS in ER+ samples (*p* = 2e-11, Fig. 3i), but was nearly not as effective in ER– samples (*p* = 0.2, Fig. 3j). We postulate that this discrepancy was because of the larger distribution of RIPSs in ER+ tumors, whereas ER– tumors have a smaller dynamic range of RIPSs (Fig. 3h).

To examine whether RIPS contributes additional prognostic information not explained by conventional clinical factors, we constructed a multivariate Cox regression model that included both RIPS as well as other important clinical factors (e.g. age, tumor size, ER status, HER2 status, lymph node, stage, and grade) as covariates. This result indicates that the RIPS provides significant additional information about patient prognosis (*p* = 9.8e-05) even after considering all these clinical factors (Table 1).

**Table 1:** The result of a multivariate Cox regression model using METABRIC database

Variable	Type	Coefficient	*p* value	Hazard ratio	95 % CI
RIPS_RAD51_	Continuous	1.8E-02	9.8E-05	1.018	1.009 ~ 1.027
Age	Continuous	6.0E-04	9.0E-01	1.000	0.992 ~ 1.009
Tumor size	Continuous	1.2E-02	5.3E-07	1.012	1.008 ~ 1.017
ER status	Binary	–1.7E-01	2.1E-01	0.845	0.651 ~ 1.097
HER2 status	Binary	3.9E-01	5.3E-03	1.470	1.122 ~ 1.926
Lymph node	Integer	9.0E-02	1.0E-15	1.094	1.071 ~ 1.119
Stage	Integer	9.2E-01	1.4E-01	0.922	0.828 ~ 1.028
Grade	Integer	9.2E-01	1.8E-01	0.922	0.939 ~ 1.409

Except RIPS_RAD51_, other important clinical factors including age, tumor size, ER status, HER2 status, lymph node, stage, grade are also considered as variables to input to a Cox regression model. RIPS_RAD51_ is significantly prognostic of survival

**Fig. 4:**
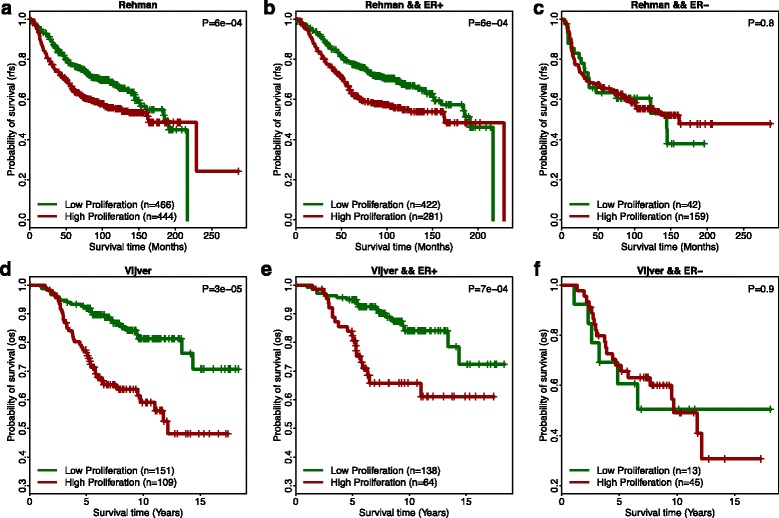
Prognosis of Ur-Rehman and Vijver breast cancer patients using RIPS_RAD51_. **a** *Kaplan–Meier plot* of RIPS_RAD51_ in Ur-Rehman database. Patients with higher cell proliferation (RIPS >0, *red curve*) shows worse survival prognosis. **b** *Kaplan–Meier plot* of RIPS_RAD51_ for ER+ patient samples in Ur-Rehman data. Patients with higher cell proliferation (*red curve*) show worse survival prognosis. **c** *Kaplan–Meier plot* of RIPS_RAD51_ for ER– patient samples in Ur-Rehman data. **d** *Kaplan–Meier plot* of RIPS_RAD51_ in Vijver database. Patients with higher cell proliferation (RIPS >0, *red curve*) shows worse survival prognosis. **e** *Kaplan–Meier plot* of RIPS_RAD51_ for Vijver ER+ patient samples. Patients with higher cell proliferation (*red curve*) show worse survival prognosis. **f** *Kaplan–Meier plot* of RIPS_RAD51_ for Vijver ER– patient samples.

We further validated the predictive value of RIPS_RAD51_ with two additional breast cancer datasets. We calculated tumor RIPS in the Ur-Rehman dataset that combines multiple independent datasets measured by Affymetrix one-channel arrays [32], as well as the Vijver dataset that was generated using only two-channel arrays [33]. In both datasets, patients with low cell proliferation (RIPS < 0) showed significantly better survival outcome than those with high HR cell proliferation (RIPS > 0). Again, the predictive power of RIPSs was greater in ER+ tumors than ER– tumors (Fig. 4).

### RIPS predicts response to neoadjuvant chemotherapy

**Fig. 5:**
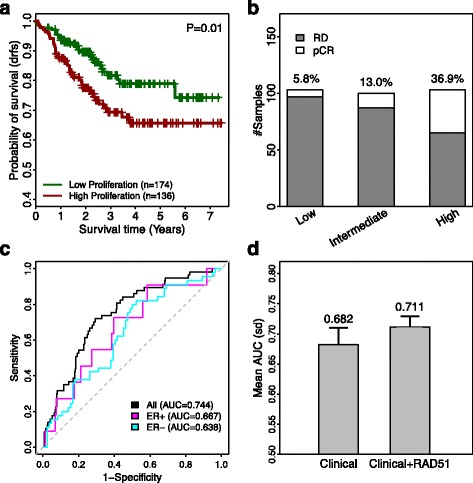
Prognosis of Hatzis discovery data using RIPS_RAD51_. **a** *Kaplan–Meier plot* comparing survival of high to low cell proliferation patients. Patients with low RIPS (*green curve*) have significantly higher survival than patients with high RIPS (*red curve*). **b** *Barplot* of the pCR rate within low, intermediate, and high RIPS groups comparing the number of RD patients (*gray*) to the number of patients achieving pCR (*white*). The pCR rate is given above each *bar*. **c** *ROC curve* calculating accuracy in classifying pCR patients. *Black curve*: All patients in Hatzis dataset (AUC = 0.744). *Magenta curve*: ER+ only Hatzis patients (AUC = 0.667). *Cyan curve*: ER– only Hatzis patients (AUC = 0.638). **d** *Barplot* comparing average AUCs from a random forest model either including RIPS_RAD51_ with clinical information or not. Mean AUC is given above each *bar. Error bars* represent standard deviation in the AUC distribution

Treatment by chemotherapeutics is broadly applied to manage many types of cancers. To discover whether RIPS predicts the responsiveness of breast cancer patients to neoadjuvant chemotherapy, we calculated RIPS of tumors included in the GSE25055 discovery dataset from Hatzis et al. [34]. This dataset contains clinical outcomes and response data from 508 patients with breast cancer treated with neoadjuvant chemotherapy. First, we found that RIPS was prognostic of survival outcome; patients with RIPS-low tumors exhibited favorable prognoses (Fig. 5a, *p* = 0.01). Second, we stratified patients into three groups based on RIPS and compared the proportions of patients that showed pCR after administration of neoadjuvant chemotherapy. As shown in Fig. 5b, the pCR rate was 5.8 %, 13.0 %, and 36.9 % in the low, intermediate, and high RIPS groups, respectively. Impressively, breast cancer samples with high RIPS were 6.4-fold more likely to respond to neoadjuvant therapy than those with low RIPS. This suggests that RIPS could be used as a biomarker to predict the responsiveness of individual breast tumors to neoadjuvant chemotherapy. Therefore, RIPS may help oncologists, in a more personalized treatment fashion, to decide whether to use chemotherapeutics to treat individual patients.

Furthermore, we used RIPS to classify patients into those who achieve pCR versus those harboring RD following neoadjuvant chemotherapy using ROC; this yielded high accuracy (AUC = 0.744, Fig. 5c). Moreover, much higher prediction accuracy was achieved in ER+ (AUC = 0.667) than in ER– breast cancers (AUC = 0.638). We constructed two Random Forest models to classify pCR versus RD samples in the GSE25055 dataset—in one model, we included both RIPS and clinical factors (i.e. age, ER status, PR status, HER2 status, stage, grade, and node status) as predictors, while in the other model we included only clinical factors. Cross-validation results indicated that utilizing RIPS improved the mean accuracy to predict tumor response to neoadjuvant chemotherapy from an AUC of 0.682 to 0.711 (Fig. 5d). We also implemented these analyses in the validation dataset (GSE25065) to ensure that our results remained consistent (Additional file 1: Figure S4).

### RIPS correlates with genomic instability in breast cancer

**Fig. 6:**
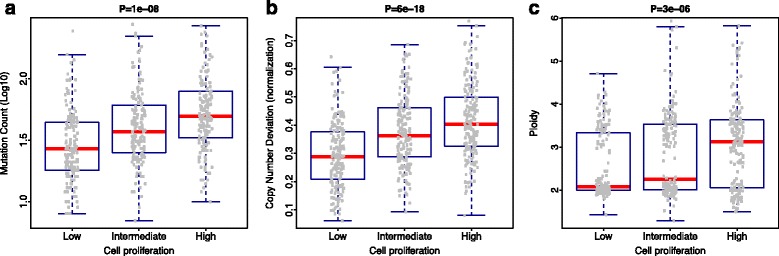
High cell proliferation correlates with high genomic instability metrics. **a** *Boxplot* of log10 transformed mutation counts in different cell proliferation groups. Each *gray spot* indicates log10 transformed mutation counts. The width of each *box* is proportional to the sample number and the *p* value represents an ANOVA calculation. **b** *Boxplot* of the CND score distributions in the three groups. **c** *Boxplot* of estimated tumor ploidy distributions in the three groups

Next, we investigated whether the calculated RIPS is able to infer genome instability. Specifically, we used three different metrics of genomic instability within The Cancer Genome Atlas (TCGA) breast cancer data [35]. First, we counted the total number of somatically mutated genes for each sample (see “Methods”). Second, based on copy number variation data we defined a metric termed CND, which is a weighted average of absolute copy number aberrations in breast cancers (see “Methods”). Finally, using the ABSOLUTE algorithm [39], we estimated the ploidy of each breast tumor. We then calculated the RIPS of all TCGA breast cancers and stratified patients into three groups based on low, intermediate, or high RIPS. We compared the three groups by total number of somatically mutated genes, CND, and ploidy. As shown in Fig. 6, these comparisons indicated significant associations between RIPS and these three metrics of genome instability. Each metric showed that cancers with higher RIPS have more unstable genomes. RIPS-high tumors retain more mutation counts than RIPS-intermediate and RIPS-low tumors (Fig. 6a, Mann–Whitney U-test, *p* = 1e-08). Similarly, RIPS-high tumors have more CND (Fig. 6b, Mann–Whitney U-test, *p* = 6e-18) and ploidy (Fig. 6c, Mann–Whitney U-test, *p* = 3e-06) than the other two tumor groups.

### Genomic alterations in RIPS-high breast cancers

TCGA provides diverse types of genomic data for breast tumors, enabling investigation of specific genomic alterations in samples with high cell proliferation. We first examined somatic mutation data to identify genes with a significant differential mutation frequency among low, intermediate, and high RIPS samples. As a result, we identified three genes using a false discovery rate (FDR) < 0.01 threshold. The most significant gene was *TP53*, which was mutated in 6.6 %, 29.1 %, and 64.3 % of tumors in the low, intermediate, and high RIPS groups, respectively (*p* = 4e-32, Chi-square test). This finding confirms our previous result that a higher RIPS_RAD51_ correlates with a higher number of *TP53* mutations in both METABRIC and TCGA datasets (Additional file 1: Figure S3). In addition, we found that *PIK3CA* and *MAP3K1* showed higher mutation rates in samples with lower RIPS (Additional file 1: Figure S5).

**Fig. 7:**
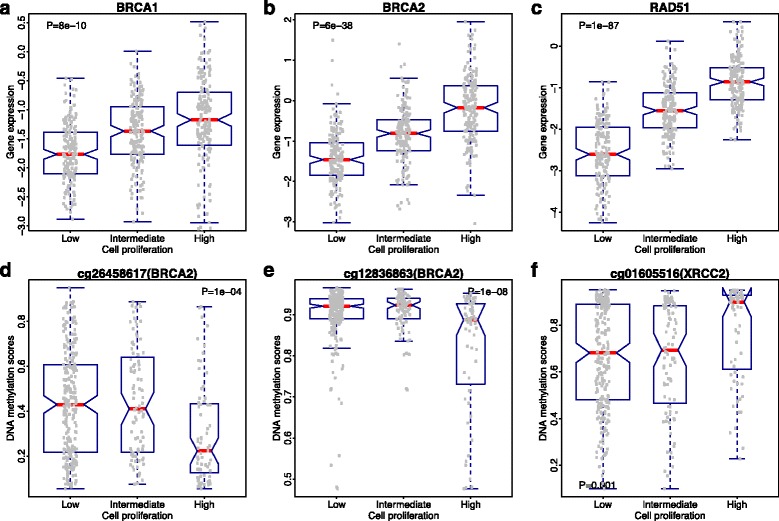
Difference of gene expression and DNA methylation in different RIPS groups. **a** *BRCA1* expressions in different RIPS groups. *Gray dots* indicate the amount of *BRCA1* expression for a particular sample. The width of the *box* is proportional to the number of samples. Reported *p* value is a result of ANOVA calculation. **b** *BRCA2* expressions in different RIPS groups. **c** *RAD51* expressions in different RIPS groups. **d** DNA methylation levels of cg26458617 in different RIPS groups. *Gray dots* indicate levels of DNA methylation of this cpg site. The width of the *box* is proportional to the number of samples. Reported *p* value is a result of ANOVA calculation. **e** DNA methylation levels of cg12836863 in different RIPS groups. **f** DNA methylation levels of cg01605516 in different RIPS groups

We then compared the mRNA expression levels of *BRCA1*, *BRCA2*, and *RAD51* in the RIPS-low, RIPS-intermediate, and RIPS-high groups in the TCGA dataset. Interestingly, all three genes showed elevated expression levels in samples with the high RIPS group (Fig. 7a–c). This indicates that higher proliferative tumor cells are associated with higher expression levels of *BRCA1*, *BRCA2*, and *RAD51*. In addition, we examined the expression levels of these three genes in hereditary cancers with *BRCA1*/*BRCA2* germline mutations versus sporadic cancers (from the Larsen et al. dataset [30]; 33 *BRCA1*-mutated cancers, 22 *BRCA2*- mutated cancers, and 128 sporadic cancers). Compared with sporadic breast cancers, the expression levels of *BRCA2* were significantly elevated in *BRCA1*-mutated samples (*p* = 5e-4, t-test), the expression level of *BRCA1* was significantly elevated in *BRCA2*-mutated cancers (*p* = 9e-5, t-test), and the expression level of *RAD51* was significantly elevated in both *BRCA1*-mutated and *BRCA2*-mutated cancers (*p* = 5e-7 and *p* = 1e-4, t-test) (Additional file 1: Figure S6). This suggests a possible negative feedback loop in the HR pathway that cells attempt to compensate the defects in *BRCA1*/*2*-mutanted tumors by upregulating other HR pathway genes.

Finally, we found that the differential expression of *BRCA1*, *BRCA2*, and *RAD51* may be partially explained by changes in promoter DNA methylation. In particular, higher levels of DNA methylation in gene promoters correlate with transcriptional silencing [45]. From TCGA data, after excluding tumors with a somatic mutation in *BRCA1*, *BRCA2*, or *RAD51*, we compared RIPS-low, RIPS-intermediate, and RIPS-high groups in terms of methylation levels in CpG sites near *BRCA1*, *BRCA2*, or *RAD51*. Our analyses identified several CpG sites with significant differential methylation levels. For example, cg26458617, a CpG site located 403 bp upstream of the transcription start site (TSS) of *BRCA2*, showed a much lower methylation level in the RIPS-high group compared to the RIPS-intermediate and RIPS-low groups (Mann–Whitney U-test, *p* = 1e-04) (Fig. 7d). In another similar example, cg12836863, a CpG site located 593 bp upstream of the TSS of *BRCA2*, also showed lower methylation levels in the RIPS-high group (Mann–Whitney U-test, *p* = 1e-08) (Fig. 7e). Integrating the compensatory mechanism proposed above (Fig. 7b and c), we found that the methylation levels of cg26458617 and cg12836863 are inversely correlated with the expression levels of *BRCA2* in samples with high cell proliferation, respectively. This suggests that hypo-methylation in the promoter region of a gene is able to cause its elevated expression. Moreover, we extended such analysis to the CpGs associated with 27 HR-related genes defined by the KEGG HR pathway [46] and also found some interesting results (Additional file 1: Table S4). For example, cg01605516 (correlated with the expression level of *XRCC2*, which is a member of the *RAD51* family [47]), a CpG site located 3527 downstream of the TSS, showed significantly higher methylation level in samples with higher cell proliferation (Mann–Whitney U-test, *p* = 0.001). We found that the methylation of cg01605516 is positively correlated with *XRCC2* expression in samples with high cell proliferation (Additional file 1: Figure S7).

## Discussion

In this study, we developed a new computational method to infer the level of cell proliferation in breast tumor samples by referring to gene expression profiles generated by RNAi-mediated knockdown of *BRCA1* or *RAD51* in MCF-10A cells. We applied our method to multiple breast cancer datasets to demonstrate that the inferred cell proliferation score can discriminate hereditary from sporadic breast cancer samples, predict prognosis and efficacy of neoadjuvant chemotherapy, and correlate with genomic instability in breast cancer.

We started our analysis from the *BRCA1* and *RAD51* knockdown profiles provided by Peng et al. [27]. As suggested by the pathway enrichment analysis, the readout of knocking down either *BRCA1* or *RAD51* affects DNA replication more than DNA repair (Additional file 1: Table S3). Moreover, the RIPSs inferred by using *BRCA1*/*RAD51* knockdown profile were positively correlated with the expression of *MKI67*, a well-known proliferation marker [42] (R = 0.81 and R = 0.78 for *BRCA1* and *RAD51*, respectively). These results suggested that the inferred RIPSs are more likely to reflect the effect of *BRCA1* or *RAD51* knockdown on reducing cell proliferation. This is consistent with previous studies that HR deficiency results in more DNA damage which delays the activation of cell cycle checkpoints and causes cell cycle arrest [4, 41].

Then, we calculated two RIPS measures using either the *BRCA1* or *RAD51* knockdown profile as a reference; however, our analyses indicated that the *RAD51* knockdown provided a better estimator of the alterations of the defective HR pathway. Compared to *BRCA1*, the *RAD51* knockdown profile not only yielded better results when predicting the prognosis of breast cancer patients and classifying BRCA mutation progeny from sporadic cancers, but was also more highly associated with DNA replication pathways as shown by gene functional annotation enrichment analysis. Besides *BRCA1* and *RAD51*, Peng et al. [27] provided the knockdown profile of *BRIT1* (GSE54269), a key gene involved in HR pathway [48], which was used to define the gene signature they used. Additionally, we repeated the survival analysis in the METABRIC dataset [31] using the *BRIT1* knockdown profiles (Additional file 1: Figure S8). In light of these results, the *RAD51* knockdown remained a better estimator of the readout of HR pathway.

We found that breast tumors with inherited *BRCA1* or *BRCA2* mutations tend to have higher RIPSs than sporadic breast tumors. Indeed, we could use RIPS to classify hereditary versus sporadic breast tumors (Fig. 2), which implied that RIPS is a robust biomarker in breast cancer. In addition, RIPS provided a computational approach to discriminate hereditary from sporadic breast tumors. We reasoned that this was possibly due to the fact that mutant *BRCA1/2* carriers exhibit dysfunctional HR pathway at the onset of carcinogenesis, whereas sporadic cancers may have somatic mutations to develop cancer. However, we noted that discrimination was imperfect; a large number of sporadic breast tumors were associated with equal or higher RIPS than breast tumors from mutant *BRCA1/2* carriers. This is consistent with the concept of BRCAness: that there exist other mechanisms that can inactivate the HR-mediated DNA repair pathway in sporadic breast cancer resulting in accelerating tumor proliferation. For example, it has been shown that BRCA1 transcription can be repressed by aberrant methylation in its promoter in sporadic breast cancer [17]. Thus, it would be useful to investigate the sporadic breast tumors with extremely low RIPS for the identification of alternate mechanisms leading to low cell proliferation.

Additionally, we found that patients with higher RIPS tended to have shorter RFS intervals, but were more likely to respond to neoadjuvant chemotherapy. This is consistent with the fact that high cell proliferation correlates with increased sensitivity to taxane chemotherapy during inhibition of microtubule disassembly [49–51]. In addition, we observed that breast tumors with high RIPS were more likely to have TP53 mutations, which renders chemotherapy less effective [52]. Thus, a combination of RIPS with TP53 mutation status might improve the prediction of response to neoadjuvant chemotherapy; tumors with high RIPS and wild-type TP53 would be likely to be more chemosensitive.

Moreover, we tested whether using the gene expression level of one gene or a small group of genes would have a similar predictive and prognostic power when compared with the complete knockdown profiles of *RAD51*. We performed these analyses in the METABRIC breast cancer dataset [31]. First, we tested the association of gene expression of 27 genes in the KEGG HR pathway [46] using a Cox regression model. We found that 11 genes were correlated with patient survival (FDR < 0.01), including *BLM*, *RAD54L*, *POLD1*, *RAD54B*, *EME1*, *XRCC3*, *RPA2*, *POLD3*, *TOP3B*, *RAD51*, and *MUS81*. These results underperformed compared to using complete knockdown profiles of *RAD51* (Additional file 1: Table S5). In addition, we performed the same analysis using the *MKI67* expression. The result suggested that though *MKI67* has been used as a biomarker for proliferation [42], its performance (*p* = 2e-15, survival difference *p* value) was less predictive than that using the complete profile of *RAD51* (*p* = 1e-18, survival difference *p* value). Moreover, we found that the inferred RIPS (AUC = 0.744) is much more predictive to the response of neoadjuvant therapy (pCR versus RD) than MKI67 (AUC = 0.668). These results suggested that using a gene set as the marker is more stable, which generates higher prediction. We further identified the 300 most upregulated and downregulated genes of *RAD51* to repeat the survival analyses. The survival analysis results from using the 300 most downregulated genes as references (*p* = 2e-16, Wald test) were similar to but slightly lower compared to using the complete profile of *RAD51* (*p* = 1e-18, Wald test). Furthermore, using the 300 most upregulated genes resulted in a much lower association with survival (*p* = 9e-06, Wald test). These suggests that the 300 most downregulated genes better reflected the actual alterations that occur when knocking down *RAD51*. Moreover, these observations demonstrate that using the complete knockdown profile is more sensitive when capturing the alterations of the pathway as it becomes ablated.

Recently, single-sample GSEA (ssGSEA) analysis has been widely used to infer the activity of pre-defined pathways in biological samples with gene expression profiles [53, 54]. However, in most cases, genes in a pathway were provided as a gene set and generally without knowing whether a gene imposes a positive or negative effect on the pathway. Furthermore, these gene sets only indicate which genes are part of the pathway without considering the magnitude of its effect. BASE [28] differs in this aspect by utilizing continuous information directly from the gene expression profile to assess the weight of each gene. In fact, we applied ssGSEA analysis to calculate the activities of several curated DNA repair or damage response pathways in breast cancer data and failed to obtain biologically meaningful results. The method we proposed in this study was special in that it calculated the tumor cell proliferation by referring to knockdown profile of genes in the HR pathway using quantitative manner, manifested from both directly and indirectly regulated genes by the pathway, and informs upregulation and downregulation. Thus, the RIPS calculated by this method can effectively reflect the readout caused by HR deficiency and can be applied as a useful biomarker for breast cancer clinical applications.

## Conclusions

Based on *BRCA1* and *RAD51* knockdown expression profiles and primary breast tumor expression data, we provide a computational method to infer the cell proliferation level for each breast cancer patient. The calculated RIPS is able to accurately reflect the cell proliferation and imply great potential in breast cancer clinical applications.

## Additional file

Additional file 1: Figure S1. The gene consistency across six different breast cancer datasets. **Figure S2.** Distinguishing hereditary and sporadic breast cancer samples using RIPS. **Figure S3.** TP53 mutation rate in different breast cancer datasets. **Figure S4.** Prognosis of the Hatzis validation dataset using RIPS_RAD51_. **Figure S5.** Mutation rate of significant genes in different cell proliferation patient groups. **Figure S6.** Gene expression of *BRCA1*, *BRCA2*, and *RAD51* in different breast cancer mutations. **Figure S7.** Gene expression of *XRCC2* in different cell proliferation patient groups. **Figure S8.** Prediction of BRIT1 in the METABRIC breast cancer dataset. **Table S1.** Breast cancer datasets used in this analysis. **Table S2.** Regulated gene lists for BRCA1 and RAD51. **Table S3.** Pathway enrichment of genes regulated by BRCA1 and RAD51. **Table S4.** HR-related genes and their DNA methylation level in RIPS-low, RIPS-intermediate, and RIPS-high groups. **Table S5.** Survival analysis results for 11 significant HR genes using their expression level. (PDF 843 kb)

## Abbreviations

AUC: Area under the curve
CDF: Cumulative distribution function
CND: Copy number deviation
GEO: Gene Expression Omnibus
HR: Homologous recombination
pCR: Pathologic complete response
RAS: Regulatory Activity Score
RD: Residual disease
RFS: Relapse-free survival
RIPS: RNA Interference derived Proliferation Score
RNAi: RNA interference
ROC: Receiver operating characteristic
ssGSEA: single-sample GSEA
TCGA: The Cancer Genome Atlas
TNBC: Triple-negative breast cancer
