# Supplementary material for: Application of RNAi-induced gene expression profiles for prognostic prediction in breast cancer
Source: Genome Med. 2016 Oct 27;8:114. doi: 10.1186/s13073-016-0363-3 (PMC5084341; doi:10.1186/s13073-016-0363-3)
Supplement: Additional file 1: Figure S1. — The gene consistency across six different breast cancer datasets. Figure S2. Distinguishing hereditary and sporadic breast cancer samples using RIPS. Figure S3. TP53 mutation rate in different breast cancer datasets. Figure S4. Prognosis of the Hatzis validation dataset using RIPSRAD51. Figure S5. Mutation rate of significant genes in different cell proliferation patient groups. Figure S6. Gene expression of BRCA1, BRCA2, and RAD51 in different breast cancer mutations. Figure S7. Gene expression of XRCC2 in different cell proliferation patient groups. Figure S8. Prediction of BRIT1 in the METABRIC breast cancer dataset. Table S1. Breast cancer datasets used in this analysis. Table S2. Regulated gene lists for BRCA1 and RAD51. Table S3. Pathway enrichment of genes regulated by BRCA1 and RAD51. Table S4. HR-related genes and their DNA methylation level in RIPS-low, RIPS-intermediate, and RIPS-high groups. Table S5. Survival analysis results for 11 significant HR genes using their expression level. (PDF 843 kb) [file 13073_2016_363_MOESM1_ESM.pdf]

Additional file 1:

Supplementary materials

Application of RNAi-induced gene expression profiles for prognostic prediction in breast cancer

Yue Wang[1,2], Kenneth M.K. Mark[2], Matthew H. Ung[2], Arminja Kettenbach[3,4], Todd Miller[2,3], Wei Xu[1], Wenqing Cheng[1], Tian Xia[1,*], and Chao Cheng[2,3,5,*]

[1]School of Electronic Information and Communications at Huazhong University of Science and Technology, Wuhan, Hubei, 430074, China.
[2]Department of Molecular and Systems Biology, Geisel School of Medicine at Dartmouth, Hanover, NH 03755, USA.
[3]Norris Cotton Cancer Center, Geisel School of Medicine at Dartmouth, Lebanon, NH 03766, USA.
[4]Department of Biochemistry, Geisel School of Medicine at Dartmouth, Hanover, NH 03755, USA.
[5]Department of Biomedical Data Sciences, Geisel School of Medicine at Dartmouth, Lebanon, NH 03766, USA.

Contents

Figure S1. The gene consistency across 6 different breast cancer datasets.

Figure S2. Distinguishing hereditary and sporadic breast cancer samples using RIPS.

Figure S3. *TP53* mutation rate in different breast cancer datasets.

Figure S4. Prognosis of Hatzis validation dataset using RIPS$_{RAD51}$.

Figure S5. Mutation rate of significant genes in different cell proliferation patient groups.

Figure S6. Gene expression of *BRCA1*, *BRCA2* and *RAD51* in different breast cancer mutations.

Figure S7. Gene expression of *XRCC2* in different cell proliferation patient groups.

Figure S8. Prediction of *BRIT1* in METABRIC breast cancer dataset.

Table S1. Breast cancer datasets used in this analysis.

Table S2. Regulated gene lists for *BRCA1* and *RAD51*.

Table S3. Pathway enrichment of genes regulated by *BRCA1* and *RAD51*.

Table S4. HR-related genes and their DNA methylation level in RIPS-low, -intermediate and -high groups.

Table S5. Survival analysis results for 11 significant HR genes using their expression level.

Figure S1. The gene consistency across 6 different breast cancer datasets. The size of each circle imply the Jaccard score of two datasets. The bigger circle, the higher Jaccard score. Purple infers high consistency and orange infers no consistency.

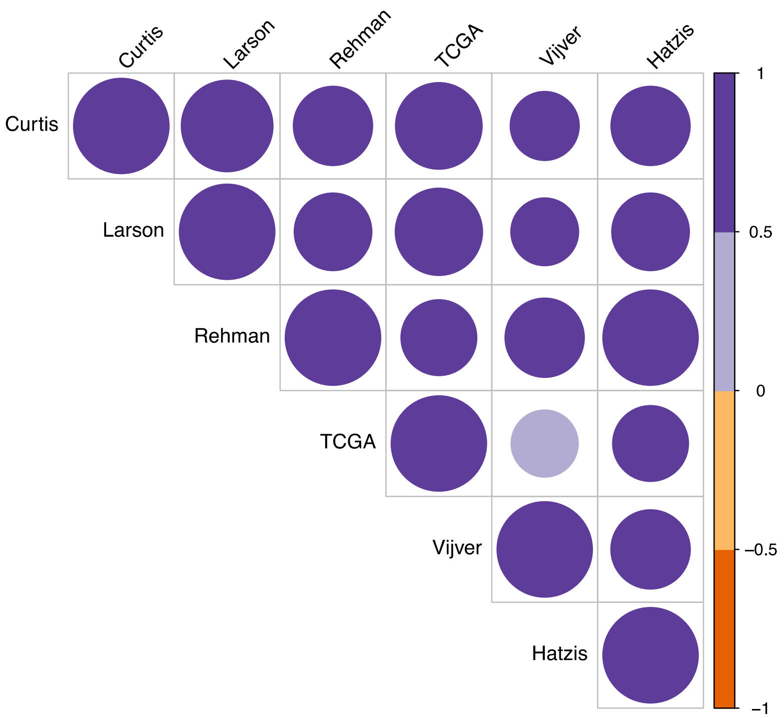

Figure S2. Distinguishing hereditary and sporadic breast cancer samples using RIPS. (A) Boxplot of inherited (both *BRCA1*- and *BRCA2*-related) cancers and nonfamilies sporadic cancer patient based on $RIPS_{BRCA1}$. The width of each box is proportional to the sample number. The p-value is a calculation of one-way analysis of variance (ANOVA). (B) Same as (A) but utilizing $RIPS_{RAD51}$.

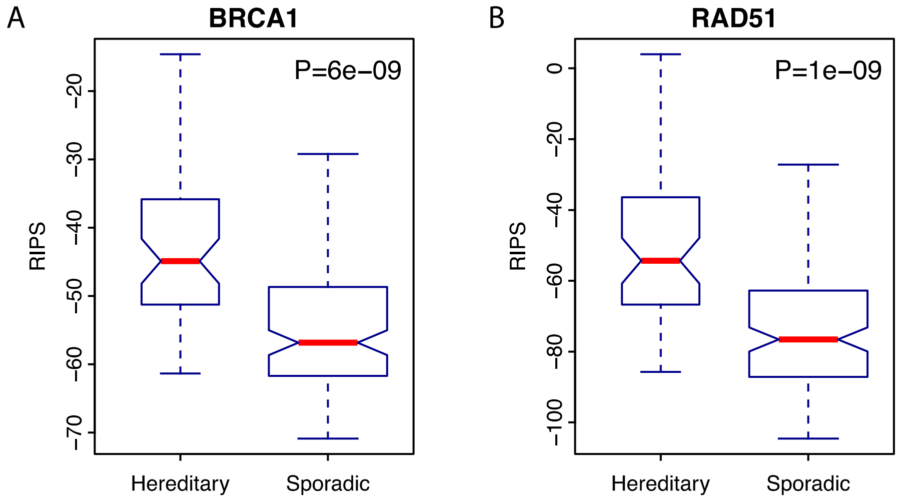

Figure S3. ***TP53*** **mutation rate in different breast cancer datasets.** (A) Using METABRIC breast cancer dataset. The grey dashed line represents the *TP53* mutation rate across all samples within the dataset. The x axis represents the average RIPS of each sliding window (here, the window size was 200). The Y axis demonstrates the *TP53* mutation rate for each window. (B) same as (A) but utilizing TCGA breast cancer dataset.

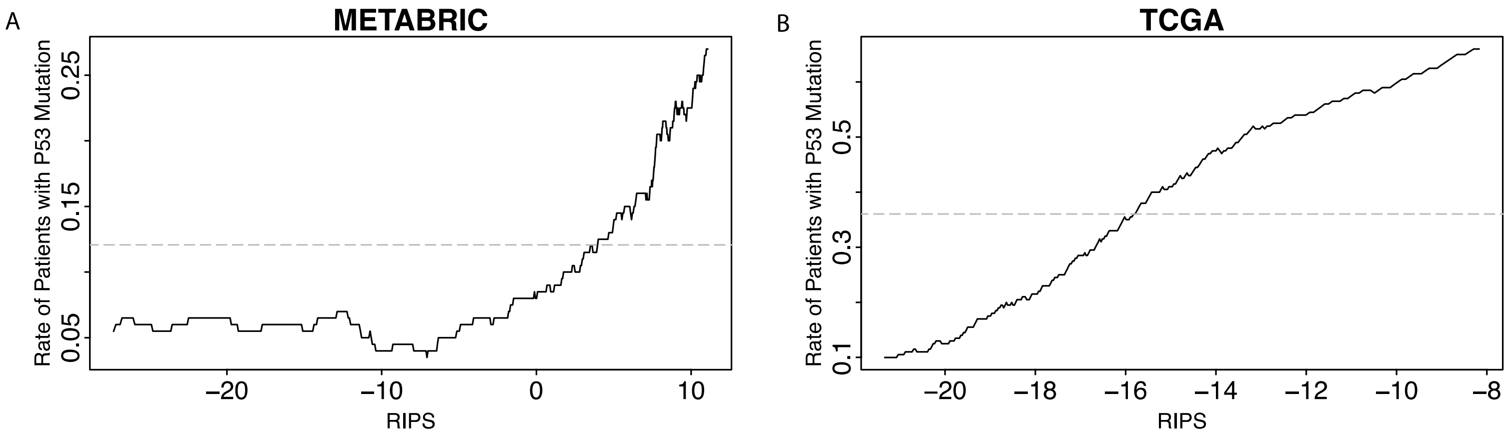

Figure S4. Prognosis of Hatzis validation dataset using $RIPS_{RAD51}$. (A) Kaplan-Meier plot of patients in Hatzis validation dataset. The green curve is patients with low RIPS and the red curve is patients with high RIPS. The difference between the two curve is significant (P=0.02). (B) Barplot of pCR% of samples within low, intermediate and high RIPS groups. The grey bar: the number of patients with RD status. The white bar: the number of patients with pCR status. The pCR% is shown above each bar. (C) ROC curve of patients with pCR status. Black curve: ROC for all patients in Hatzis dataset (AUC=0.653). Magenta curve: ROC for patients with ER+ status (AUC=0.66). Cyan curve: ROC for patients with ER- status (AUC=0.581). (D) Barplot comparison for prediction of pCR using either clinical information or clinical information plus $RIPS_{RAD51}$. Average of AUC calculated from 10-fold cross validation is shown above the bar. The vertical line in each bar represents the standard deviation of the 10 AUCs.

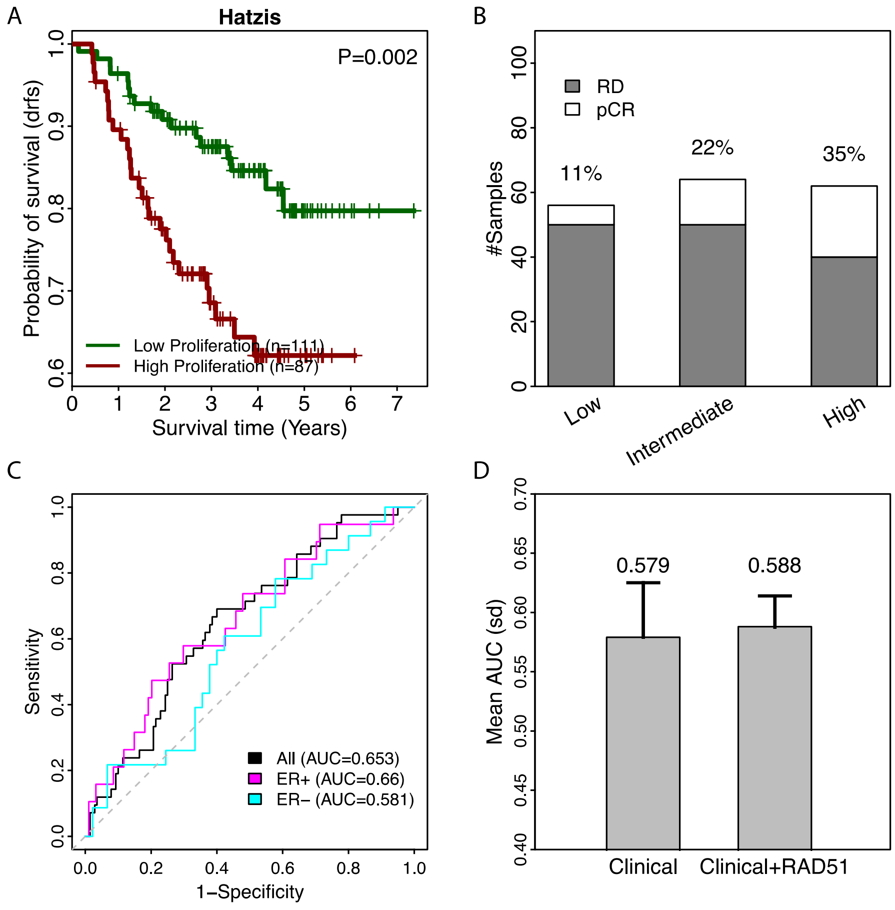

Figure S5. Mutation rate of significant genes in different cell proliferation patient groups. Red, yellow and blue bars represent low, intermediate and high RIPS tumor groups, respectively. Chi-squared test P values were presented with stars to show the difference between three groups. * indicated p value < 0.01 and ** indicated p value < 1e-10.

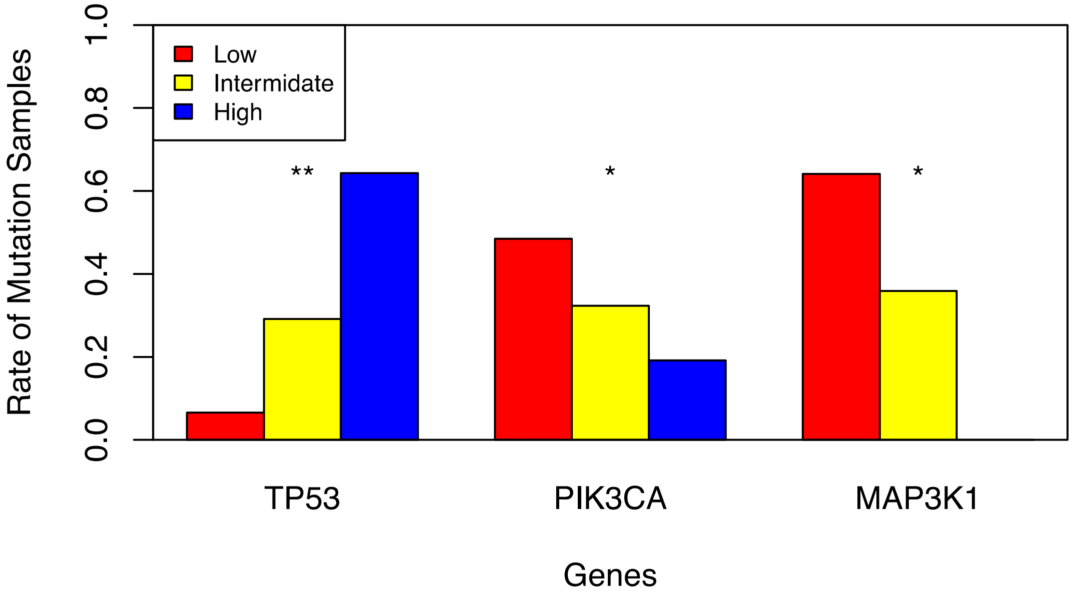

Figure S6. Gene expression of *BRCA1*, *BRCA2* and *RAD51* in different breast cancer mutations. (A) Boxplot of *BRCA1* expression in *BRCA1*-mutant, *BRCA2*-mutant and sporadic breast cancer samples. Each grey spot indicated a *BRCA1* expression score. The width of each box was proportional to the sample numbers. (B) Same as (A) for *BRCA2* expression data. (C) Same as (A) for *RAD51* expression data. T-test P values were presented using stars to show the difference between groups. ** indicated p value < 5e-04.

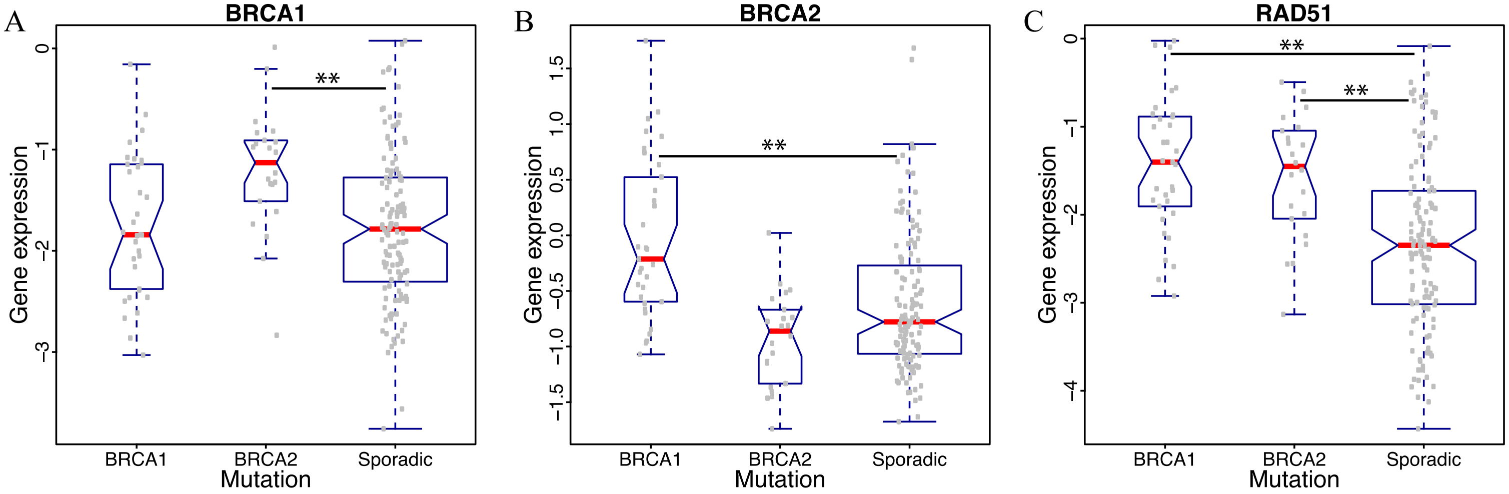

Figure S7. Gene expression of *XRCC2* in different cell proliferation patient groups. Boxplot of *XRCC2* expression in breast cancer samples with different cell proliferation. Each grey spot indicated a *XRCC2* expression score. The width of each box was proportional to the sample numbers. The p value was calculated by one-way analysis of variance (ANOVA).

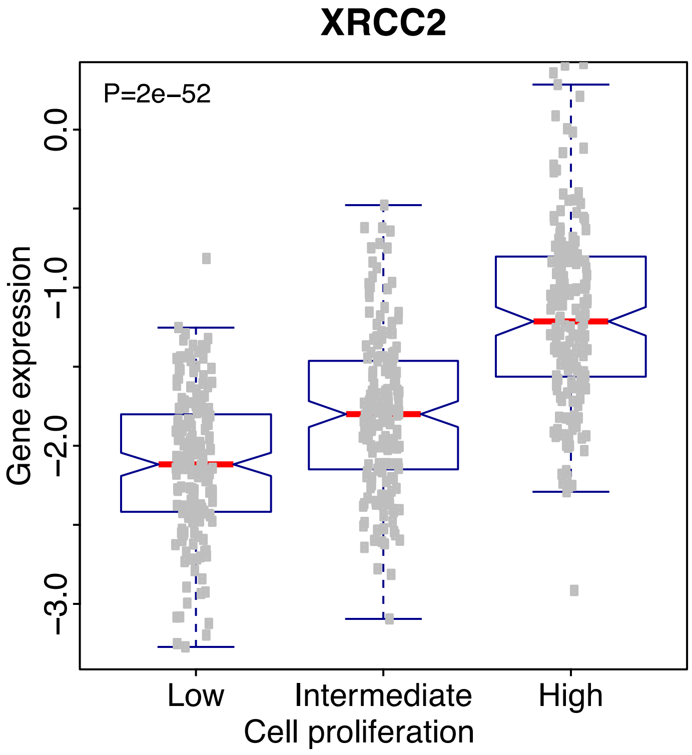

Figure S8. Prediction of *BRIT1* in METABRIC breast cancer dataset. Kaplan-Meier plot comparing survival of high to low cell proliferation patients. Patients with low RIPS (green curve) have significantly higher survival than patients with high RIPS (red curve).

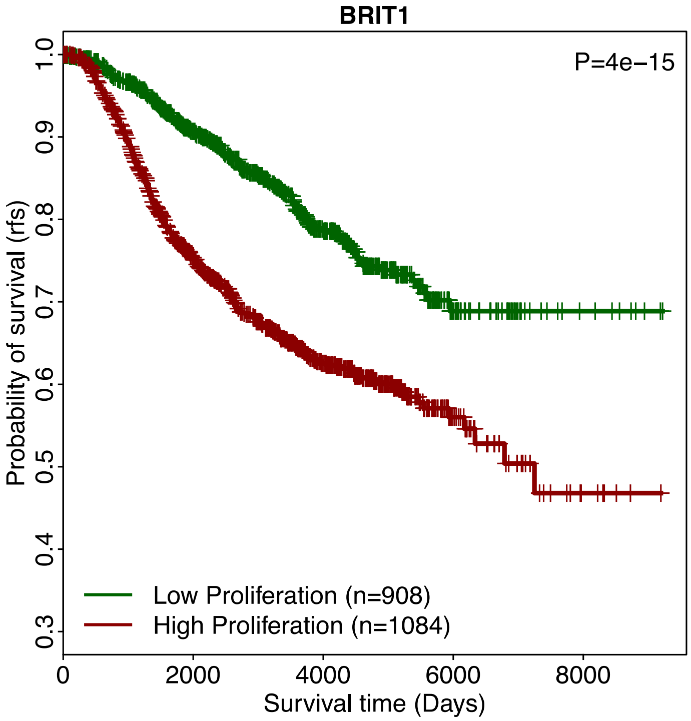

Table S1. Breast cancer datasets used in this analysis.

Source	PubMed ID	Accession ID	Number of samples	Outcome	Platform	Mutation Types
Larsen et.al	PMID:23704984	GSE40115	183		GPL15931 & two channel	Somatic & Germline
Ur-Rehman et.al	PMID:23756628	GSE47561	910	RFS	one channel	
Vijver et.al	PMID:12490681		260	OS	two channel	
Curtis et.al	PMID:22522925	EGAS00000000083	1992		one channel	
Hatzis et.al	PMID:21558518	GSE25066	310	DMFS	GPL96 & one channel	
Cancer Genome Atlas Network	PMID:23000897		972		two channel	

Table S2. Regulated gene lists for *BRCA1* and *RAD51*.

Types	Gene List
BRCA1_Up	MTMR9, PLAC8, SNCG, PITPNM1, MUC20, APP, FUT3, SLC35A3, SEMA3B, ZFAND2B, ITGB2, TIGA1, PRDM1, SLC7A11, GPR1, DSG3, SNAI2, LOC201175, DUSP5, C16orf68, FAM83E, IL1A, ST6GALNAC2, HIST2H2AA3, PHLDA3, CYP4B1, FAM24B, STK19, ISG20, PPP1R15A, LOC728285, ARRDC4, FLOT1, GRAMD2, B4GALT4, COX7A1, C3, PTGDS, KLHL9, DAPP1, ZNF260, GCNT1, DUSP14, HSF1, VASN, C1orf24, PPP1R13L, LOC643150, LOC651116, DFNA5, POU3F1, HLA-A, PRODH, CKB, LPIN1, PROS1, TOB1, CA5B, MAP3K13, RALGPS1, AGPAT7, PPAPDC1B, LOC285359, LRP11, DDIT3, NDFIP2, PTGS2, KIAA1267, TAF15, SIL1, TRPM4, CHIC2, THOC6, DPH3, C10orf116, ARL5B, IBRDC3, GPRC5A, EDN1, C9orf23, GSS, WASF2, SAMSN1, DDEFL1, TSC22D2, CBLB, INHBB, THBS1, TP53INP2, IFI6, PHLDA1, KLK10, PJA2, TNFRSF12A, LIPG, ZNF329, GABRP, DHDH, ANKRD13A, CRABP2, SLC9A8, B3GALT4, STAT2, BAMBI, TJP1, ZNF226, LCN2, UNC5B, CCDC90B, LOC130576, KIAA0513, GADD45A, CRIP2, GTPBP2, TERF1, C5orf38, CES2, TNFRSF14, TINF2, TXNIP, WBP4, NR1D2, ATP10B, C21orf7, UBR4, RRAGD, HCST, TLN2, CLASP1, C6orf205, KIAA1434, C9orf150, RARRES3, AXL, KLHDC9, C1orf54, PLEKHA7, IDUA, MAP1LC3B, ATF6, BAIAP2L1, CDH3, LIMA1, FOXD1, LOC554203, FAM43A, PLD5, SLC35D2, NFIL3, DAPK1, ADM, HES2, PEX11G, SCEL, NAPRT1, YPEL5, KCNB1, MARVELD2, SPRR1B, CHMP4C, GCNT3, CEACAM6, XDH, RNASE7, DGCR2, NPC1, C4orf34, TP53INP1, CRYL1, CLMN, CDA, CD68, ITPKC, HSD17B8, C20orf102, MGC42630, SPRR1A, C9orf66, C16orf52, PLK2, C19orf33, RNF24, PVRL2, CDKN1C, KLK5, GADD45B, WBSCR27, LOC730820, PHLDB2, CYP4F11, PIK3IP1, C20orf149, HIST1H3D, TGM1, KRT13, LOC646463, BMP2, PLCD3, CD59, TSPAN31, CLDN23, C10orf97, IRF6, MYO6, CRYAB, CYB5R1, PEX7, MRPS18A, FOLR3, FSTL3, ZFAND1, ZBTB43, CHPF, CPE, HIST2H2BE, KIAA1539, LOC100008589, DPF2, DCBLD2, KLF2, CYP4F3, LOC652767, TMEM142C, LCE1B, FLII, VARS2, RMND5B, PRNP, C10orf73, IFIT2, NFE2L1, FEZ2, NDE1, LRRC8A, FAM46A, TCN2, EPHB3, TMEM8, PTPRH, UAP1, DUSP8, MIF4GD, INPP5D, SERPINE1, C20orf121, LYRM5, MIA, ZNFX1, ISGF3G, KLK7, SLC45A4, IGFBP3, CLDN8, LOC729843, MYL5, EVI5L, TSPAN1, TCEAL4, TMPRSS11B, LYRM1, TCEAL3, VRK2, C10orf47, SDSL, SYTL1, RBCK1, FLJ90086, SESN2, ZCWPW1, LHPP, DOCK5, LAMB2, VTCN1, FAM86C, MED19, SH3TC1, MGC4172, C14orf179, NTN4, HIST1H1C, UFM1, C11orf68, SLC31A2, SNX12, WFS1, RND3, CLIP4, IFI44, TMEM42, POLR3GL, ENSA, COBLL1, 7A5, C12orf39, MAP2, HLA-E, SLC2A12, C6orf48, ADAMTS6, SERPINB7, DCN, CCDC71, HIST2H2AC, ELF3, PLCD1, CTSO, SERPINB13, CDH4, BTG2, KRT34, KLF5, RPAIN, MAL2, PNMA1, PSCA, D4S234E, TMEM27, PHYH, USMG5, DKK1, DNAJB9, HSPA5, ST6GALNAC5, FAM63B, FLJ45831, SOCS1, FAIM3, STX19, BTG1, PPCS, RAG1AP1, LOC153222, PRSS22, CTH, C10orf65, HYPE, SFRS7, CLIC5, C20orf19, KLF4, RRAS, ARHGEF3, TUFT1, WIPI1, CNN3, CPA4, ATP6V1G1, COL22A1, TLCD1, ALDH6A1, LEMD1, OBFC2A, C1orf74, SNX16, PRIC285, SLC27A1, TRIB3, PNPLA2, DOCK8, OBFC1, C8orf70, CFLAR, POSTN, VPS28, XPC, NXN, HSPB8, C9orf103, PFN4, HIST1H2AC, ID2, SERTAD1, FUT2, SPRR2A, SNX24, LOC338758, VIL2, OKL38, CLIC3, PRSS8, NCF2, F3, LOC729252, PRSS23, CD55, BBS1, GSN, EMP1, CPLX1, C1orf50, ZNF91, ZNF185, TMC4, CRCT1, POLD4, LOC146439, HCP5, SLC16A4, CBS, C14orf128, WDR13, CD24, ADAMTS9, PPL, C16orf74, PAWR, C14orf28, ALDH1A3, UBAP1, FAM46B, BCAS4, C19orf22, ZNF25, HRASLS2, CYP4F12, NDRG1, FAM129A, SPRR3, FAM134B, MAPK8IP3, CBLC, ATP6V1B1, DNAJC15, HSD11B2, BCMO1, CPNE4, RNF141, DENND2C, 39510, ABCG1, ACSL3, AGXT2L2, AIM1L, ALDH3B1, ALS2CL, AMY2A, ANK3, ANXA11, ARHGAP23, ARSD, ARTN, ATP2B4, ATP5S, BAIAP2, BCL2L1, BLOC1S2, C14orf78, C15orf48, C1orf116, C21orf81, C9orf85, CCD C80, CCNB1IP1, CDCP1, CDKN1A, CEACAM1, CHMP2A, CLYBL, CMTM7, COL17A1, CORO2A, CRELD1, CST6, DHRS9, DLC1, EPS8L1, FBLN1, FBXW7, FLJ20444, FLRT3, FRYL, FST, FYN, GALE, GOSR2, GPR110, GPSM1, GRHL3, HDAC9, HIST1H2BD, IFIT1, IL1RN, ITGA3, JUP, KCNG1, KLK6, KLK8, KRT23, LAMB3, LOC136288, LOC554223, LOC644322, LOC644739, LOC647859, LOC650517, LOH11CR2A, LRRFIP2, MAPK10, MB, METRNL, MME, MTMR3, NGFRAP1, NOTCH2NL, NRG1, OAS2, OASL, PCDH1, PCK2, PCNXL2, PHACTR3, PITPNC1, PLAG1, PLEC1, PRDX5, RAET1G, RARRES1, RASA1, RASSF6, RGS20, RIOK3, RPS6KA2, SCFD1, SDCBP2, SHC1, SIRT5, SLC16A5, SLFN5, SLMO1, SPTBN1, SRPK2, STX16, STX5, SULT2B1, SYTL2, TCEA1, TGM2, TIMP2, TOM1L2, TRIOBP, UGT1A6, UPP1, VRK3, WWP2, ZNF585A
BRCA1_Down	CDT1, CCNE2, KDELC2, FHOD1, CBR3, POLD1, ERF, CABLES1, HSD3B7, FZD2, HAPLN3, FAM136A, METTL3, FANCI, SLC39A10, LOC645304, PES1, C16orf59, HIBCH, CCL20, RAD54L, LOC732371, SLC46A3, NUF2, MGC61598, LOC644617, GABRA2, MTBP, MCOLN2, CDCA2, RAB31, PAQR4, CSRP2, PGAM1, CENPJ, PDSS2, LCP1, MGC87042, TGFBI, EPHA1, CHEK1, DNA2L, LOC645351, GSDMDC1, TUBB, C14orf106, SLC25A19, SNORD30, IL8, HBA2, TNS3, SLC25A13, OAF, CYP4X1, NUP85, CHAF1A, DKFZp686O24166, POLR2L, NRM, C3orf21, F12, MND1, SAMM50, AKR1B10, ZC3H12A, CRYZ, DNAJA1, DPYSL2, RPL28, PBK, GALNTL4, NUP205, CHAF1B, TFRC, PRKAR1B, C15orf42, OLAH, NAGPA, ALDH1B1, EPDR1, VSNL1, SLIT2, ADCY3, CYFIP2, SNAPC4, LRP8, LOC643287, RRM2, DFFB, NSMCE4A, LEF1, DPYSL3, LOC643997, MLF1IP, C2orf33, PAXIP1, C5orf13, CXorf6, MCM2, GGH, MASTL, CXCL2, TEAD2, CPVL, RPUSD1, PRICKLE2, NCAPD3, CDCA5, TOMM40, AURKB, TNFAIP8L1, TAF5, ALG8, ACOX2, KIF2C, HSPA8, DEFB1, LOC402644, PARP1, VCAN, NCOA7, ST6GALNAC4, TBC1D4, IL27RA, MAP6D1, PTDSS1, FLJ20273, MRTO4, CNTNAP2, TMEM20, NSDHL, MYO1G, LOC653381, PHB, PI3, CYP1B1, RAC3, WDR79, GPD1L, NFIX, SNORD35B, PRIM1, SLC39A8, ASF1B, C8orf32, KIF20A, LOX, C21orf63, SELI, FABP5, SFRS2, CHST4, CA8, POLA2, TGFBRAP1, OGT, FAM54A, SLC19A1, FAM81A, IL6, LOC732007, SNORA62, LARS2, TROAP, SLC27A2, SAP30, EPHX1, GBP1, ABCA6, AKR1B1, AYTL2, C9orf140, TMEM166, RECQL4, TWF2, PDSS1, FZD3, EDG3, FHL3, AHSA1, SERPINB3, DKFZp762E1312, CMAH, ACTG1, SPR, ACPL2, C6orf150, LIG1, GEMIN6, TNIP3, H2AFZ, PLEKHF1, USP13, LMNB2, PDZK1IP1, NT5DC2, GSG2, SHCBP1, BLM, SRPX, CMBL, FBXO5, UBE2T, ANKRD10, COBL, MYB, TRIM47, CCNB1, AKR1C3, SEMA6A, CORO1A, CYB5A, DNAH2, GLCE, PSEN2, MT1G, SOSTDC1, NNMT, TFAM, NEIL2, SDPR, POLM, LOC441454, HOXC8, MT1F, MGC23985, ABHD7, TNC, GMNN, LOC91431, CA12, RANBP1, MLZE, PAPPA, BLMH, L2HGDH, SLC29A1, TACC3, RFC4, LYSMD2, SLC27A5, DHCR24, SLC45A3, C6orf32, NUSAP1, TAF6L, C1orf112, TMEM171, FNTB, PRC1, MSH6, PRR6, GATM, TSPAN9, HERC5, S100A8, LOC653994, RFP, MRPL16, SERTAD4, KCTD14, CABC1, MELK, UBXD6, KIAA0101, KNTC1, GNB4, HMGB3, MPP1, MMD, TATDN2, SORD, CHST12, TIMELESS, PLK1, PDXP, C13orf3, ZDHHC23, MAT2A, MSH2, CDCA3, TMEM118, SLC1A3, PLAT, TMEM70, ANLN, ALS2CR13, CD14, POLQ, CYP27B1, RFX5, CDC7, ESPL1, LPXN, TUBA1A, FZD4, RUSC1, WDR67, KCNIP3, RHOBTB3, CDC42EP4, HNRPM, POLR2F, IRAK2, RERG, C18orf17, PCOLCE2, DDX39, LOC389901, PLCXD1, CD3EAP, RBM33, CALB2, C18orf55, SMC2, BANF1, TUBD1, CUL1, DLG7, TMEM48, LOC342541, YWHAG, SERPINH1, FASTKD1, SH2B3, TMEM55A, MCF2, PTTG1, HOXA5, CSK, GINS3, LOC85389, DHX15, RPS26, FEN1, NUP62CL, DEPDC6, S100A7, NEIL3, STC1, ACPP, UGT8, MLLT11, OIP5, SRP9, PTMA, DNMT1, CD70, TMEM46, ZMIZ2, NETO2, RAB8A, GNB1L, LOC441019, FHOD3, C10orf119, EFHD2, DHFRL1, NOD2, ECE2, FAM119A, XTP3TPA, BID, EVI2B, HDAC4, MAFB, EMP3, LOC399942, DOCK11, C16orf57, ACY1L2, TERC, GLA, PKMYT1, CCDC138, XRCC6BP1, NY-SAR-48, TOR3A, POP1, GJB2, STRA13, IDE, SFPQ, CHRNA5, RBBP7, GEMIN4, POLRMT, ELL3, CD47, ALG6, RRM1, PRKCA, NUP37, TAF4B, LOC653752, C20orf82, FABP4, NFKBIA, MRGPRX3, OR7E91P, FECH, POLE2, NAB1, NCAPD2, MT1X, NOX5, LOC646197, LOC148915, DCC1, PDK3, SAC3D1, ANKRD38, CFD, E2F2, MSX1, MAD2L1, C8orf55, FIGNL1, FAM111A, LOC644774, PUS7, HPSE, UTP18, LOC387934, THAP11, TUBB2C, MGC16075, SUV39H1, C9orf152, FAM83D, GCN5L2, TMEM56, SERPINB4, ARPM1, DHFR, C6orf167, HELLS, TUBA4A, PLCE1, NUP210, COPS7B, CREBZF, FASN, ATP6V0E2, TMEM14A, MTHFD1, POLR3H, UHRF1, CCNA2, LPL, HCN3, CXCL1, SLC25A12, TTK, FRAT2, SERPINA3, SLITRK5, HSPA1A, TMEM145, PLK4, C11orf82, DPY19L1, TUBB3, 76P, TMEM158, LOC201725, ESCO2, DNAJB6, KIF11, SNRPD3, DPYD, RPA1, MMP9, AYTL1, TRIP13, PKN3, HSPA14, HMGCS1, ALOX5AP, EXTL2, C11orf75, NUDT21, MOSC1, ZNF511, C16orf33, CXCR4, POLR3K, CCDC109B, CCNB2, GLT8D2, DEPDC1, TARBP1, WDR76, ADA, HSPE1, SLC25A10, FDPS, C7orf27, RAVER2, VRK1, TK1, TYMS, PAQR5, MCM3, C14orf145, LHX2, GINS4, PADI4, SNCAIP, KIF14, TMEM180, GUCY1A3, FSCN1, HSD17B2, CBR1, CARHSP1, GINS2, PPIF, NGEF, RDH16, RNASEH2A, HMHA1, PLEK2, PHCA, SLC7A6, CENTD3, SOX18, DDX54, KIAA1199, PRMT6, NR1H3, LY6D, SPC25, TRPS1, DEPDC1B, MRPS2, MCM5, ASPM, NUP107, LOC199800, AADAT, ABCF2, ABR, ACCN2, ACSL5, AKR1C2, AKR1C4, ALDH3A2, ATP11C, ATP6V1C1, AZI1, B3GALNT1, BCL11B, BRCA1, BRI3BP, BTBD11, C12orf48, C14orf130, C17orf41, C1QTNF6, C3orf37, CACYBP, CBX2, CCNE1, CDCA7, CDK2, CHN1, CSE1L, CSTF3, CTSC, CYB5R2, DHX9, DLL3, DONSON, DUT, EXO1, EZH2, FAM13A1, FAM20C, FARP1, FUT8, GJA3, GPER, GPM6B, GTF2I, GYPC, HEATR2, HEATR3, HNMT, HSPA4, HSPCAL3, IL17RB, IL1R2, INSIG1, KHK, KIAA1147, KRT6B, KYNU, LOC286467, LOC442582, LOC643224, LOC647000, LOC649679, LOC653226, MANEAL, MCM7, MEST, MGC40489, MLKL, MPST, MRE11A, NEDD4, NFATC1, NSUN5C, OTUB2, PCNA, PKIA, PNKD, PPIL5, PSRC1, PTPLAD1, PUS1, RAD54B, RFC2, RFC3, RFC5, RGS10, ROBO1, SAA1, SCARA3, SKP2, SLC35F3, SLC43A3, SLTM, SNRP70, ST5, STEAP1, STX2, SULT1A1, TBRG4, TMEM97, TPM2, TRAF5, TSPAN18, TSPAN3, TTC13, TWIST2, UNG, WDHD1, WDR90, ZC3HAV1
RAD51_Up	SELM, GADD45G, MGP, SPATA7, C11orf35, ECH1, SEMA3B, ZMAT3, ADCY9, TESK2, TIGA1, ZCCHC11, GPR1, DSG3, EIF4B, KIAA0427, TSHZ2, DSP, TMEM159, TSPAN10, ALDH4A1, ABLIM3, APCDD1, LEPREL1, VAV3, MAPKBP1, WDR54, C16orf68, TP53AP1, LOC728910, UBXD1, ST6GALNAC2, C10orf26, FAM116B, HIST2H2AA3, PHLDA3, LOC92017, STK19, PPP1R15A, ARRDC4, RIN1, LGALS7, LOC644760, PTK7, ZNF358, PNPLA7, LFNG, CITED2, ZNF337, DMXL1, PTGDS, KLHL9, PPP1R14C, KRT14, ZNF260, ANKRD19, DUSP14, SPATA18, KIAA1467, KIAA0913, DFNA5, ZNF20, PRODH, CKB, PROS1, CLCC1, TOB1, EPHA4, ZNF615, C10orf57, CIRBP, PRMT2, LOC644799, BLCAP, COL4A6, KLHL12, LRP11, HTRA1, PTPRZ1, LOC645553, TIGD7, MTSS1, DDIT3, C8orf40, PTGS2, ZNF559, KIAA1267, ESR1, PEX11B, FLJ35220, ZNF581, TRPM4, WBP5, C10orf116, CD109, ZNF606, ZNF404, FTH1, IHPK2, GSS, C11orf70, ZNF425, NPAL3, ZFP90, TECT1, DDEFL1, CSF1R, TP53INP2, IFT140, SPATA20, IFI6, CTNNBIP1, KLK10, CAMP, RECK, ZNF329, ZBTB20, ANKRD13A, C3orf54, PTPLB, STAT2, TJP1, LOC728006, UNC5B, CCDC90B, CROT, LOC130576, KIAA0513, PDCD6IP, SLC47A2, GADD45A, CRIP2, TERF1, ADRB2, C5orf38, LOC388610, CES2, TNFRSF14, TINF2, TXNIP, TMEM43, ATP10B, C21orf7, CCT6B, RRAGD, TLL1, FLJ37464, HCST, UST, TLN2, CLASP1, ABHD4, VAT1, KIAA1434, C9orf150, AXL, KLHDC9, C1orf54, ZNF706, ALDH3A1, SH3BGRL, ADHFE1, IDUA, MAP1LC3B, TBC1D9, CDH3, LIMA1, LOC554203, LTB4DH, C6orf85, SLC35D2, NFIL3, MST1, C20orf108, PAK6, DAPK1, EAF2, ADM, EPHX2, PLEKHM1, HES2, PEX11G, APLP2, PAPLN, NAPRT1, YPEL5, KCNB1, SPRR1B, ANXA4, SSBP2, EDEM2, CYBRD1, RNASE7, TCP11L1, DGCR2, LOC654096, LOC440928, C4orf34, TP53INP1, SPSB1, RDH10, CRYL1, CD86, CD68, ERMAP, HSD17B8, CCDC53, C20orf102, CEBPG, IGBP1, SLC29A3, C19orf33, MAP2K5, CDKN1C, KLK5, BNIP3L, WDR26, PIK3IP1, C20orf149, ZFP36, TULP4, PCDHB4, BMP2, PLCD3, OTUD1, CEL, CSNK2A2, CBX4, CD59, TSPAN31, IRF6, APTX, GJA1, LOC730273, COL5A2, NOTCH1, CRYAB, TPP1, CYB5R1, RNF39, FOLR3, NPHP3, FSTL3, CCDC92, ZBTB43, TEX2, CHPF, CPE, HIST2H2BE, MN1, ID3, MFAP5, LOC100008589, MAPRE3, BBOX1, SH3PX3, CYP4F3, PLEKHG4, TMED10, TMEM142C, LCE1B, PPP1R3C, DIXDC1, KRT16, NTF5, PCMTD1, LOC88523, FLJ10986, LOC124446, C10orf73, HS1BP3, DOPEY1, NFE2L1, NDE1, APBB3, MACROD1, HERC3, EPHB3, WDR47, COL4A5, F2R, TBC1D20, SH3PXD2A, CA11, DLL1, ZNF446, INPP5D, SERPINE1, C20orf121, LYRM5, PTPRM, ZNFX1, KLK7, SPOCK1, GHDC, GTF2IRD2P, PAQR7, SAT2, LOC729843, MYL5, EVI5L, GOLGB1, TCEAL4, PSAP, TCEAL3, PI4K2A, SDSL, MGC102966, SYTL1, COL7A1, RBCK1, SESN2, ZCWPW1, ALOX15B, UBE3B, LHPP, LAMB2, VTCN1, FAM86C, KRT6C, JARID1B, BEST2, MFGE8, SH3TC1, C14orf179, MAPK6, HIST1H1C, UFM1, HCFC1R1, TAOK1, SIRPA, SLC9A9, SLC31A2, WFS1, IRS1, MAN2B1, RND3, CLIP4, RRAGC, POLR3GL, ACAD11, PIR, COBLL1, OLFML2A, SEMA3F, LOC654191, EGR1, PIGV, C7orf10, ADH1A, HLA-E, DSC3, IL20RB, C6orf48, CDH13, RNF150, SCPEP1, BBS2, LASS6, SERPINB7, PCMTD2, PDCD4, ANKRD24, DCN, MAN2B2, HIST2H2AC, C9orf52, PLCD1, RIN2, FLRT2, SERPINB13, BTG2, AGRN, KRT15, KLHL3, TMEM134, SYNC1, LOC653513, GSTA4, D4S234E, LOC388681, ZNF395, TMEM27, GAS1, PHYH, DNAJB9, HSPA5, ST6GALNAC5, CALCOCO1, DNAJB1, TRAPPC6A, BTG1, GHR, PPCS, RAG1AP1, LOC153222, IFT74, C10orf65, ANXA8, CLN5, NUDT2, WDR41, KLF9, TRIM22, C20orf19, KLF4, LOC400027, ARHGEF3, WIPI1, NPL, VIM, LETMD1, PCDHB12, TGFBR3, CPA4, SEPP1, ALDH6A1, LEMD1, PRKAB2, P2RY5, RAB22A, SLC27A1, TRIB3, NUDT18, DOCK8, BMPR2, ZNF181, POSTN, VPS28, XPC, HUWE1, YPEL3, NXN, HSPB8, C9orf103, SIDT2, PFN4, CCDC104, CAMSAP1L1, HIST1H2AC, ZFP37, ZNF766, SERTAD1, CCPG1, FLJ41481, FLJ41603, MCC, P4HA2, LOC338758, ZBTB4, SMPDL3A, CLIC3, PRSS8, GRINA, MAP3K12, LOC729252, PRSS23, BLVRB, KIAA0746, EID2B, CCDC51, ETNK2, CST3, BBS1, SESN1, MANBA, RPL37, GSN, GPNMB, SLC29A4, GPC1, AMZ2, KRT5, DNAL4, C1orf50, OR7E156P, ZNF91, IQCG, ZNF185, TMC4, POLD4, IQGAP1, THBS3, C6orf130, C14orf128, PERLD1, PARC, PPL, C14orf45, SFN, C14orf28, NDST1, FAM46B, BCAS4, ZNF25, EHD2, HRASLS2, VAMP5, LOC643161, NDRG1, ATP6V1E2, CYP3A5, TDRD9, ZNF211, FAM134B, C9orf119, HSD11B2, C9orf9, RNF141, DENND2C, NINJ1, LOC730877, KIAA1881, SPTLC3, DFNB59, AAK1, ABCA2, ABCA5, ABCG1, ABLIM1, ACSL3, ADPRHL1, ADSSL1, AIM1L, ALDH3B1, ALS2CL, AMY2A, ARSD, ATP2B4, ATP5S, ATP6V1G2, ATRN, BAIAP2, BCL2L1, BCL6, C14orf78, C1orf116, C21orf33, C4orf18, CCN B1IP1, CDKN1A, CHMP2A, CLYBL, CMIP, COL17A1, CRELD1, CSNK1A1, CSNK1G1, DLK2, DNAJC12, DSEL, DST, DUSP22, ERGIC1, EVC, FBLN1, FBXO2, FBXW7, FLRT3, FNIP1, FOXO3, FXYD3, FYN, GLUL, GPSM1, HIST1H2BD, HOXC4, IL11RA, IL17RC, ITGA3, JUB, JUP, KCNG1, KIAA1641, KITLG, KLK6, KLK8, LAMB3, LOC136288, LOC400566, LOC400578, LOC554223, LOC650517, LOH11CR2A, LPHN3, LRRC29, LRRFIP2, LYPD5, MAF, MAPK10, MME, MYLK, NEBL, NGFRAP1, PARD6G, PCBP4, PCDH7, PCDH9, PCDHA1, PHACTR3, PIK3R1, PLAG1, PLD3, PLEC1, PLXNB2, RAET1G, RAPGEF1, RASSF6, RFNG, RIOK3, RNF128, RPS6KA2, RRAGB, RTN1, SDCBP2, SERINC3, SHC1, SLC16A5, SLC3A2, SLC7A8, SLFN5, SMG1, SMPD1, SNCA, SRPK2, STARD5, STX16, STX5, SULF2, TCEA2, THSD1, TRIM29, TSC1, TSC22D3, TSPYL2, UGT1A6, VRK3, WNK1, ZMYND8, ZNF136, ZNF585A, ZNF658, ZNF658B
RAD51_Down	CDT1, CCNE2, DCK, FHOD1, C15orf23, CBR3, POLD1, THEX1, LOC400451, CABLES1, HSD3B7, FUT3, C14orf80, ATRIP, HMGB2, FAM136A, METTL3, FANCI, LOC642031, TAF1A, PLAU, LOC645304, C16orf59, CCL20, POLR2J, CENPI, RAD54L, LOC732371, SLC46A3, SAAL1, NUF2, MGC61598, C6orf66, TJP3, GABRA2, MTBP, MCOLN2, CDCA2, RAB31, PAQR4, LOC643438, PGAM1, LOC649604, CENPJ, TNFRSF6B, C14orf156, C5orf34, COX7A1, LCP1, CDC20, MGC87042, EPHA1, DHTKD1, CENPF, CHEK1, DNA2L, GSDMDC1, TUBB, C14orf106, SLC25A19, SNORD30, IL8, SQRDL, HBA2, SLC25A13, SAA4, OAF, DDAH1, CENPM, CYP4X1, GLRB, CHAF1A, TSPAN13, RAD51AP1, ATOX1, PHLDA2, F12, MND1, AKR1B10, CHCHD1, IFITM2, PBK, NUP205, CHAF1B, PRKAR1B, TMEM71, C15orf42, OLAH, LSM6, EPDR1, SRPX2, SLIT2, ADCY3, DUSP6, LRP8, LOC643287, ARHGDIB, RRM2, NUDCD1, BCYRN1, DFFB, SNRPF, SERPINB1, NSMCE4A, LEF1, DPYSL3, LOC643997, SH2D4A, MLF1IP, ADRA1B, POP7, PAXIP1, LRRC26, AURKA, MCM2, BIRC3, GGH, MASTL, DKFZp434K1815, CXCL2, IER3, C13orf27, TEAD2, STAMBPL1, CPVL, LOC440731, NCAPD3, CDCA5, TOMM40, AURKB, TNFAIP8L1, TAF5, ALG8, COL8A1, ACOX2, KIF2C, THBS1, HSPA8, DEFB1, LOC402644, CCDC58, RFWD3, SEMA4D, NCOA7, TBC1D4, IL27RA, FLJ20273, MRTO4, CNTNAP2, TMEM20, DDEF1, PLAUR, HS3ST3A1, MYO1G, LOC653381, SH3BP1, PHB, PI3, WDR79, GPD1L, SNORD35B, PRIM1, PAQR8, LOC646723, SLC39A8, CCDC99, LY6E, ASF1B, KIF20A, C21orf63, XRCC3, FABP5, SFRS2, CHST4, CA8, POLA2, LOC653972, FAM54A, RHOV, SLC19A1, LOC648682, FAM81A, IL6, TROAP, ELOVL6, KRTCAP3, SLC27A2, SAP30, LOC642897, GBP1, SLC24A6, AKR1B1, AYTL2, C9orf140, TNFAIP3, RECQL4, PDSS1, EDG3, AHSA1, TNIP1, SERPINB3, DKFZp762E1312, C10orf125, ACTG1, KRT80, SPR, ACPL2, C6orf150, H2AFV, DENND2D, AGMAT, GEMIN6, SNHG3-RCC1, TNIP3, H2AFZ, MICB, PLEKHF1, LMNB2, EHF, ADAM8, IRF1, PDZK1IP1, ACAT2, NT5DC2, NOLA1, GSG2, SHCBP1, CDCA8, ALDH1A1, BLM, LETM1, FBXO5, ANKRD39, MNS1, UBE2T, COBL, MYB, C21orf45, CKLF, RPL39L, MPHOSPH1, CCNB1, AKR1C3, LOC653879, LOC342897, SEMA6A, MREG, CORO1A, CYB5A, DNAH2, GLCE, MARCKSL1, MT1G, CASC5, SOSTDC1, NNMT, TFAM, FOXM1, RPA3, POLM, TMEM16A, LOC441454, HOXC8, MT1F, MGC23985, SERPINB8, LSM3, CKS1B, ABHD7, SYTL4, TNC, GMNN, LOC91431, CENPN, RANBP1, EXOSC9, MLZE, PAPPA, GPD2, L2HGDH, SLC29A1, TACC3, RFC4, SLC27A5, SLC45A3, MAP3K5, THOP1, NUSAP1, C1orf112, SYNCRIP, TMEM171, KIAA1524, SAA2, PRC1, MSH6, PRR6, GATM, C14orf151, HERC5, S100A8, LOC653994, DBNDD2, MRPL16, SERTAD4, SGOL1, KCTD14, ORC6L, MELK, UCHL5, KIAA0101, SLC16A9, KNTC1, GNB4, HMGB3, MMD, SORD, TIMELESS, PLK1, PDXP, C13orf3, CENPK, MSH2, RPL29, CDCA3, ATAD3A, PLAT, HS6ST2, ANLN, CD14, TYRO3, POLQ, CYP27B1, SLC5A6, CDC7, ESPL1, LPXN, TUBA1A, RUSC1, WDR67, KCNIP3, CYBA, CDC42EP4, IRAK2, RERG, CGN, PCOLCE2, CEP55, DDX39, C17orf60, PLCXD1, CD3EAP, CALB2, CNKSR3, SMC2, SDF2L1, BANF1, TUBD1, ACTL6A, MAPK13, DLG7, LOC441241, TMEM48, LOC342541, TUBB4Q, LOC162073, DKFZP564J0863, NCAPG, CKAP2L, SH2B3, SLC25A40, DHRS3, MCF2, PTTG1, GINS3, LOC85389, DHX15, N6AMT1, RPS26, FEN1, CKS2, CHTF18, S100A7, NEIL3, STC1, ACPP, FAM107B, UGT8, PLS1, OIP5, DNMT1, SRGN, TMEM46, ZMIZ2, ENG, NETO2, GNB1L, GABPB2, LOC441019, FHOD3, C10orf119, EFHD2, TIPIN, FOXRED1, NOD2, ECE2, FAM119A, XTP3TPA, BID, EVI2B, C6orf173, DAB2, LYAR, HDAC4, EMP3, SEMA3A, LOC399942, DOCK11, ACY1L2, GLA, FOXA1, PKMYT1, CCDC138, XRCC6BP1, NY-SAR-48, POP1, UCK2, U2AF2, GLIPR1, GJB2, STRA13, IDE, SFPQ, CHRNA5, GEMIN4, GTSE1, C18orf24, BAIAP2L2, NUP37, C20orf82, NFKBIA, MRGPRX3, GBP2, POLE2, NCAPD2, MT1X, NOX5, C15orf52, IL1B, LOC653505, CHORDC1, DCC1, PDK3, CFD, E2F2, MSX1, MAD2L1, C8orf55, FIGNL1, ENC1, LOC644774, PUS7, LOC387934, TUBB2C, LYN, SUV39H1, C9orf152, HMMR, FAM83D, OSBP2, UCRC, FKBP4, NR4A2, TMEM56, LOC400759, SERPINB4, DHFR, C6orf167, LOC650832, CTPS, HSP90AB4P, HELLS, TUBA4A, SNORD25, NUP210, CREBZF, ATP6V0E2, TMEM14A, STAT4, MTHFD1, FGFBP1, SMTN, ASB9, UHRF1, CCNA2, LRRC59, LPL, C1orf135, CENPO, CXCL1, TTK, COTL1, GCLM, SERPINA3, SLITRK5, TMEM145, PLK4, C11orf82, NUDT19, ARHGAP18, DPY19L1, HMGB1, TUBB3, 76P, TMEM158, ESCO2, P704P, SHFM1, KIF11, SNRPD3, MAG1, DPYD, RPA1, PTGER4, MMP9, FYB, TRIP13, PKN3, HSPA14, ALOX5AP, C11orf75, SRPK1, NUDT21, MOSC1, DNAJC9, KIF4A, ANKRD22, TUBA1B, ZNF511, C16orf33, S100P, CENPA, KRT81, CXCR4, POLR3K, CCDC109B, PPIH, CCNB2, ITGA1, CA13, GULP1, DEPDC1, WDR76, GALK1, HSPE1, SLC25A10, FDPS, C7orf27, LOC168474, IFITM3, VRK1, ANKRD32, TK1, TYMS, LOC340598, PAQR5, MESP1, MCM3, C14orf145, LHX2, GINS4, SNCAIP, KIF14, GSTO1, DUSP4, TMEM180, GUCY1A3, LOC731049, HSD17B2, MYO5C, HMG1L1, CARHSP1, BTBD14A, GINS2, PPIF, NGEF, LOC440145, RDH16, TTF2, LBR, LOC650428, RNASEH2A, HMHA1, PLEK2, NPAL1, CYP26B1, PHCA, CENTD3, DDX54, KIAA1199, NR1H3, SPC25, DEPDC1B, FKSG30, MCM5, ASPM, NOLA3, NUP107, TUBB2A, AADAT, ABCF2, ABR, ACSL5, ADAM19, AKR1C2, AKR1C4, ANGPTL4, ANXA2, ATP5G1, BCL2L12, BOLA2, BRCA1, BRI3BP, C12orf48, C15orf48, C17orf41, C1QTNF6, C5orf25, C9orf100, CACYBP, CBX2, CBX3, CCNE1, CCT6AP1, CDC2, CDC25A, CDCA7, CDK2, CEACAM1, CREB5, CYB5R2, DENND1A, DIAPH3, DLL3, DONSON, DUT, ETV5, EVI2A, EXO1, EXOC6, EZH2, FAM20C, FANCA, FANCB, FARP1, FLJ20186, FST, GDPD3, GPER, GTF2I, HEATR2, HNMT, HPCAL1, HSPCAL3, ID1, IL15, IL17RB, IL1R2, INSIG1, KDELR3, KIAA1147, KIF23, KPNA2, KRT6B, KYNU, LOC286467, LOC442582, LOC643224, LOC643300, LOC644380, LOC647000, LOC649214, LOC649679, LOC653820, LOC727761, LOC727848, LONRF3, MANEAL, MAP2K3, MCM7, MEST, MGC40489, MLKL, MRE11A, NFATC1, NLF2, NOL5A, NUP35, OAS1, OTUB2, PARVB, PBEF1, PCNA, PGAM5, PKIA, PPIL5, PRIM2A, PRKAG2, PSRC1, PTPLAD1, PTPN22, PUS1, RAD54B, RARRES1, RBBP8, RBL1, RFC2, RFC3, RFC5, S100A4, SAA1, SH3KBP1, SLC43A3, SNRPB, STEAP1, TBRG4, TEAD4, THOC4, TMEM97, TMEPAI, TMPO, TMPRSS3, TSPAN18, TTC13, TUBG1, UBE2C, UNG, WDHD1, WDR4, WDR90, ZWILCH

Table S3. Pathway enrichment of genes regulated by *BRCA1* and *RAD51*.

Gene Group	Term	Count	PValue	List.Total	Pop.Hits	Pop.Total	Fold.Enrichment	Bonferroni	Benjamini	FDR
**BRCA1_up**	Androgen and estrogen metabolism	4	2.70E-02	91	37	5085	6.04	9.51E-01	9.51E-01	2.66E+01
	p53 signaling pathway	5	3.16E-02	91	68	5085	4.11	9.71E-01	8.29E-01	3.05E+01
	Steroid hormone biosynthesis	4	4.71E-02	91	46	5085	4.86	9.95E-01	8.30E-01	4.20E+01
	Metabolism of xenobiotics by cytochrome P450	4	8.93E-02	91	60	5085	3.73	1.00E+00	9.24E-01	6.52E+01
	Systemic lupus erythematosus	5	9.75E-02	91	99	5085	2.82	1.00E+00	8.95E-01	6.86E+01
**BRCA1_down**	DNA replication	14	2.71E-13	115	36	5085	17.20	3.36E-11	3.36E-11	3.13E-10
	Mismatch repair	9	1.85E-08	115	23	5085	17.30	2.29E-06	1.15E-06	2.13E-05
	Cell cycle	14	3.38E-06	115	125	5085	4.95	4.19E-04	1.40E-04	3.91E-03
	Base excision repair	8	9.47E-06	115	35	5085	10.11	1.17E-03	2.93E-04	1.09E-02
	Nucleotide excision repair	7	3.92E-04	115	44	5085	7.03	4.75E-02	9.68E-03	4.52E-01
	Pyrimidine metabolism	9	1.20E-03	115	95	5085	4.19	1.38E-01	2.44E-02	1.37E+00
	Pathogenic Escherichia coli infection	6	8.59E-03	115	57	5085	4.65	6.57E-01	1.42E-01	9.48E+00
	Homologous recombination	4	2.39E-02	115	28	5085	6.32	9.50E-01	3.13E-01	2.44E+01
	Folate biosynthesis	3	2.40E-02	115	11	5085	12.06	9.51E-01	2.85E-01	2.45E+01
	Terpenoid backbone biosynthesis	3	4.33E-02	115	15	5085	8.84	9.96E-01	4.22E-01	4.00E+01
	Gap junction	6	4.86E-02	115	89	5085	2.98	9.98E-01	4.30E-01	4.38E+01
	One carbon pool by folate	3	4.87E-02	115	16	5085	8.29	9.98E-01	4.03E-01	4.38E+01
	Primary bile acid biosynthesis	3	4.87E-02	115	16	5085	8.29	9.98E-01	4.03E-01	4.38E+01
	PPAR signaling pathway	5	6.82E-02	115	69	5085	3.20	1.00E+00	4.90E-01	5.58E+01
	Steroid hormone biosynthesis	4	8.32E-02	115	46	5085	3.84	1.00E+00	5.37E-01	6.33E+01
**RAD51_up**	Metabolism of xenobiotics by cytochrome P450	5	2.11E-02	91	60	5085	4.66	8.91E-01	8.91E-01	2.12E+01
	Drug metabolism	5	2.35E-02	91	62	5085	4.51	9.15E-01	7.09E-01	2.33E+01
	Steroid hormone biosynthesis	4	4.71E-02	91	46	5085	4.86	9.93E-01	8.12E-01	4.17E+01
	Focal adhesion	8	6.41E-02	91	201	5085	2.22	9.99E-01	8.22E-01	5.23E+01
	Arginine and proline metabolism	4	6.67E-02	91	53	5085	4.22	9.99E-01	7.62E-01	5.38E+01
	Axon guidance	6	7.75E-02	91	129	5085	2.60	1.00E+00	7.53E-01	5.94E+01
	Systemic lupus erythematosus	5	9.75E-02	91	99	5085	2.82	1.00E+00	7.82E-01	6.82E+01
**RAD51_down**	DNA replication	15	5.37E-15	111	36	5085	19.09	6.02E-13	6.02E-13	6.05E-12
	Cell cycle	17	6.76E-09	111	125	5085	6.23	7.64E-07	3.82E-07	7.67E-06
	Mismatch repair	8	3.37E-07	111	23	5085	15.93	3.81E-05	1.27E-05	3.83E-04
	Nucleotide excision repair	7	3.23E-04	111	44	5085	7.29	3.59E-02	9.09E-03	3.66E-01
	NOD-like receptor signaling pathway	7	2.05E-03	111	62	5085	5.17	2.07E-01	4.54E-02	2.31E+00
	Pyrimidine metabolism	8	4.23E-03	111	95	5085	3.86	3.81E-01	7.67E-02	4.70E+00
	Base excision repair	5	6.48E-03	111	35	5085	6.54	5.20E-01	9.96E-02	7.11E+00
	Oocyte meiosis	8	9.35E-03	111	110	5085	3.33	6.54E-01	1.24E-01	1.01E+01
	Progesterone-mediated oocyte maturation	7	1.03E-02	111	86	5085	3.73	6.91E-01	1.22E-01	1.11E+01
	p53 signaling pathway	6	1.53E-02	111	68	5085	4.04	8.25E-01	1.60E-01	1.61E+01
	PPAR signaling pathway	6	1.62E-02	111	69	5085	3.98	8.42E-01	1.55E-01	1.69E+01
	Homologous recombination	4	2.18E-02	111	28	5085	6.54	9.17E-01	1.87E-01	2.21E+01
	Pathogenic Escherichia coli infection	5	3.41E-02	111	57	5085	4.02	9.80E-01	2.60E-01	3.25E+01
	Epithelial cell signaling in Helicobacter pylori infection	5	5.88E-02	111	68	5085	3.37	9.99E-01	3.87E-01	4.97E+01

Table S4. HR-related genes and their DNA methylation level in RIPS-low, -intermediate and -high groups.

CpG sites	Closest_TSS_gene_name	Distance_closest_TSS	Mean_Meth_RIPS_Low	Mean_Meth_RIPS_Inter	Mean_Meth_RIPS_High	T-test_Pvalue_Low_High	T-test_Pvalue_Low_Inter	T-test_Pvalue_Inter_High
cg26458617	BRCA2	-403	0.49	0.44	0.30	3.45E-07	1.86E-01	2.49E-04
cg12836863	BRCA2	-593	0.92	0.90	0.80	1.27E-06	7.25E-02	4.28E-05
cg01430588	RAD51C	-195	0.61	0.59	0.48	1.30E-06	4.92E-01	6.10E-05
cg25339112	RAD51C	-202	0.74	0.72	0.63	2.08E-06	1.75E-01	2.71E-04
cg00453717	POLD3	41001	0.92	0.91	0.89	6.55E-06	8.43E-03	1.29E-02
cg13042487	XRCC3	430	0.21	0.23	0.10	7.28E-06	5.38E-01	1.53E-05
cg16270633	BLM	-3748	0.79	0.77	0.72	8.50E-06	1.35E-01	1.50E-03
cg20864326	XRCC2	1828	0.84	0.82	0.74	8.89E-06	6.64E-02	1.59E-03
cg01605516	XRCC2	3527	0.63	0.66	0.78	9.93E-06	2.73E-01	1.42E-03
cg02194129	XRCC3	10510	0.82	0.78	0.74	1.37E-05	2.84E-02	4.29E-02
cg08196608	RPA1	74568	0.89	0.87	0.81	1.38E-05	8.03E-03	3.13E-03
cg14172849	XRCC3	10564	0.80	0.77	0.73	1.78E-05	1.62E-02	6.02E-02
cg01663440	RAD52	2735	0.83	0.82	0.76	2.43E-05	2.07E-01	5.11E-03
cg26693701	RPA1	88536	0.92	0.92	0.89	2.92E-05	5.47E-01	2.34E-04
cg18211249	RPA1	77187	0.86	0.85	0.77	3.21E-05	5.78E-01	2.32E-04
cg26377508	RPA1	77354	0.87	0.87	0.79	3.45E-05	8.20E-01	8.55E-05
cg17258364	RPA1	77270	0.92	0.91	0.84	3.88E-05	8.35E-02	1.08E-03
cg06788856	RPA1	88697	0.95	0.95	0.93	4.22E-05	2.43E-01	6.49E-04
cg22635541	RPA1	75301	0.64	0.66	0.51	5.28E-05	5.44E-01	1.48E-05
cg17724365	NBN	-109	0.06	0.05	0.05	7.17E-05	8.56E-02	1.11E-02
cg09741519	RPA1	77503	0.86	0.87	0.75	1.28E-04	7.12E-01	7.25E-05
cg01173485	POLD3	-735	0.88	0.87	0.82	1.67E-04	1.76E-01	4.84E-03
cg07918226	RAD54B	-17	0.06	0.05	0.05	1.89E-04	6.30E-01	1.37E-03
cg03719475	RAD52	5940	0.96	0.96	0.95	2.01E-04	3.05E-01	3.35E-03
cg23193616	XRCC3	4293	0.45	0.47	0.54	3.17E-04	5.39E-01	4.23E-03
cg16291854	POLD1	8157	0.94	0.93	0.93	5.57E-04	4.49E-01	3.05E-03
cg19197440	RPA1	77997	0.79	0.78	0.70	6.14E-04	6.59E-01	2.60E-03
cg02110529	RAD51C	-266	0.77	0.76	0.70	6.62E-04	6.99E-01	3.85E-03
cg13257157	RAD54B	329	0.03	0.03	0.03	6.62E-04	8.63E-01	2.80E-03
cg10443159	RPA3	-81020	0.75	0.79	0.83	6.96E-04	1.37E-01	6.38E-02
cg25858132	RPA1	75399	0.50	0.55	0.36	9.32E-04	2.37E-01	4.35E-05
cg09706243	POLD4	-582	0.13	0.16	0.18	1.00E-03	1.83E-02	1.59E-01
cg24362026	SHFM1	-29426	0.58	0.60	0.69	1.08E-03	6.63E-01	6.02E-03
cg10643407	RAD50	-92	0.05	0.05	0.05	1.30E-03	6.99E-03	2.31E-01
cg19146577	POLD2	-8661	0.75	0.78	0.80	1.35E-03	2.80E-02	2.06E-01
cg26323655	RAD54B	495	0.78	0.78	0.69	1.47E-03	9.44E-01	1.98E-03
cg01381637	POLD1	735	0.86	0.86	0.85	1.56E-03	1.68E-01	8.43E-02
cg26885816	POLD3	-616	0.84	0.83	0.77	1.70E-03	5.99E-01	7.62E-03
cg05488279	RPA1	86498	0.66	0.65	0.75	1.95E-03	6.03E-01	8.44E-04
cg27170268	XRCC3	10128	0.87	0.83	0.77	1.96E-03	1.05E-01	1.11E-01
cg25305774	RAD54B	-10677	0.51	0.49	0.64	2.39E-03	6.44E-01	1.04E-03
cg18433268	RAD54B	304	0.05	0.04	0.04	2.40E-03	5.09E-01	1.23E-02
cg27091703	RAD54B	-85	0.05	0.05	0.05	2.55E-03	4.97E-01	2.90E-02
cg23436779	RAD51C	-132	0.09	0.10	0.07	2.55E-03	7.54E-01	1.09E-03
cg18263559	TOP3B	6445	0.94	0.93	0.90	2.68E-03	3.49E-01	2.99E-02
cg26262057	MRE11A	2	0.04	0.04	0.04	2.72E-03	8.12E-01	4.56E-03
cg10104687	RAD52	221	0.65	0.67	0.70	3.49E-03	4.01E-01	3.76E-02
cg10347949	XRCC3	6075	0.88	0.88	0.91	3.51E-03	8.91E-01	1.03E-02
cg07997877	RPA1	77581	0.84	0.84	0.77	3.80E-03	7.21E-01	1.04E-02
cg18597188	XRCC3	3153	0.37	0.44	0.44	3.86E-03	2.92E-03	9.69E-01
cg04496177	RAD54B	589	0.08	0.08	0.07	4.46E-03	5.24E-01	2.90E-02
cg10656845	POLD2	3327	0.93	0.93	0.92	4.56E-03	7.54E-01	5.89E-03
cg23639841	RAD54B	-6	0.02	0.02	0.02	5.11E-03	3.90E-01	6.71E-02
ch.7.313144R	RPA3	-69223	0.07	0.07	0.06	5.65E-03	4.62E-01	4.35E-02
cg19366479	POLD1	-3940	0.74	0.77	0.80	6.62E-03	2.50E-01	1.47E-01
cg01086369	POLD1	-2219	0.99	0.99	0.99	6.89E-03	1.58E-01	2.97E-01
cg07154931	RAD54B	111	0.05	0.05	0.04	7.66E-03	3.54E-01	7.20E-02
cg15612927	RAD52	621	0.04	0.04	0.04	7.72E-03	4.65E-01	4.62E-03
cg23369670	XRCC3	9879	0.78	0.74	0.70	7.79E-03	1.40E-01	2.29E-01
cg08157964	BRCA2	245	0.01	0.01	0.01	8.59E-03	7.36E-03	8.08E-01
cg12385370	RAD52	5961	0.95	0.94	0.94	9.17E-03	2.74E-02	6.37E-01
cg04600982	RPA1	58736	0.95	0.95	0.95	9.21E-03	1.23E-01	2.88E-01
cg27017251	TOP3B	2594	0.88	0.90	0.91	9.48E-03	7.67E-02	3.79E-01
cg12383621	RPA3	22627	0.82	0.85	0.85	9.98E-03	2.25E-02	6.60E-01

Table S4. Survival analysis results for 11 significant HR genes using their expression level.

Gene	Survival analysis		Cox regression model	
	P vaule	Q value	P vaule	Q value
BLM	6.25E-14	1.62E-12	1.11E-16	2.89E-15
RAD54L	3.84E-11	4.99E-10	1.49E-13	1.94E-12
POLD1	1.87E-08	1.62E-07	3.91E-11	3.39E-10
RAD54B	7.26E-08	4.72E-07	7.52E-08	4.89E-07
EME1	1.49E-06	7.73E-06	9.22E-07	4.79E-06
XRCC3	7.84E-06	3.40E-05	1.37E-06	5.94E-06
RPA2	1.15E-04	4.27E-04	1.94E-04	6.31E-04
POLD3	6.21E-04	2.02E-03	8.45E-04	2.20E-03
TOP3B	3.52E-03	1.02E-02	3.55E-04	1.03E-03
RAD51	4.33E-03	1.13E-02	1.52E-04	5.63E-04
MUS81	1.78E-02	3.85E-02	3.99E-03	9.44E-03
